# TurboBlom: A light and resilient key predistribution scheme with application to Internet of Things

**DOI:** 10.1371/journal.pone.0295190

**Published:** 2024-03-20

**Authors:** Majid Khabbazian, Reihaneh Safavi-Naini, Ahmad Shabani-Baghani

**Affiliations:** 1 Department of Electrical and Computer Engineering, University of Alberta, Edmonton, Alberta, Canada; 2 Department of Computer Science, University of Calgary, Calgary, Alberta, Canada; Jaramogi Oginga Odinga University of Science and Technology, KENYA

## Abstract

In the Internet of Things (IoT), there are often devices that are computationally too constrained to establish a security key using traditional key distribution mechanisms such as those based on the Diffie-Hellman key exchange. To address this, current solution commonly rely on key predistribution schemes (KPSs). Among KPSs, the Blom scheme provably provides the highest resilience against node capture attacks. This, however, comes at high computational overhead, because the Blom scheme requires many multiplications over a large finite field. To overcome this computational overhead, we present TurboBlom, a novel amendment of the Blom scheme. TurboBlom circumvents the need for field multiplications by utilizing specialized generator matrices, such as random zero-one matrices. We demonstrate that, through this approach, TurboBlom can significantly reduce the computational overhead of the Blom scheme by orders of magnitude. In our next key finding, we demonstrate that TurboBlom offers a level of resilience against node capture that is virtually on par with the Blom scheme. Notably, we prove that the gap between the resilience of the two schemes is exponentially small. These features of TurboBlom (i.e., low computational overhead and high resilience) make it suitable for computationally constrained devices. Such devices exist in abundance in IoT, for example, as part of Low Power and Lossy Networks (LLNs). To demonstrate a sample application of TurboBlom, we show how to use it to enable sender authentication in the Routing Protocol for LLNs (RPL), a standard routing protocol for IoT.

## 1 Introduction

To ensure the confidentiality and integrity of data exchanged, it is crucial that network devices are able to establish security keys between themselves. These keys are used to safeguard the privacy of user information and prevent various attacks such as protocol manipulation [1], and guarantee the integrity of the data [2]. In networks such as LLNs and IoT, however, there are often devices that are computationally too constrained to establish a security key using traditional key distribution mechanisms such as those based on the Diffie-Hellman key exchange. These devices include Class 1 of constrained devices defined in RFC 7228 [3]. Devices in this class are capable enough to run a protocol stack specifically designed for constrained nodes (e.g., the Constrained Application Protocol (CoAP) over UDP [4]) and communicate with nodes employing a full protocol stack with the help of a gateway. They are, however, unable to implement a full protocol stack such as Transport Layer Security (TLS), and related security protocols according to [3].

Given enough time, devices in higher classes in RFC 7228 have the potential to run traditional key exchange schemes. For example, experimental results in [5] show that their proposed Diffie-Hellman Protocol requires, on average, 54 seconds to establish a key between two TelosB IoT devices. It’s important to note that the higher execution time of traditional key exchange schemes in these devices exposes them to denial of service attacks where, for instance, an adversary would consume their time/power resources by repeatedly initiating key exchanges with them.

An alternative to the traditional key distribution schemes is key predistribution scheme (KPS), commonly considered for networks with constrained devices [6, 7] In a KPS, a trusted entity preloads each device/node with one or more secret keys before the node is deployed in the network. After deployment, a pair of nodes in the network use their preloaded secret keys to establish a pairwise key and consequently secure their communications.

The simplest key predistribution strategy is to use a single shared key over the entire network. This scheme is the most efficient KPS in terms of memory usage, as each node stores only a single secret key. On the negative side, the scheme is the most vulnerable KPS to node corruption as a single corrupted node (e.g., a node captured and compromised by an adversary) compromises the security of the entire network, as the entire network uses one identical security key.

An alternative strategy is to preload each node with many secret keys; one key for every other node in the network. This way, any node can securely communicate with any other node in the network using a unique secret key. This KPS is the most resilient scheme against node corruption/capture. The scheme is, however, the least efficient with regards to memory (memory requirement linearly increases with the number of nodes in the network). In addition, the scheme requires every existing node to securely receive a new secret key each time a new node is added to the network (the new keys are needed for secure communication of the existing nodes with the newly added node). The above shortcomings are not desired particularly in large-scale networks.

There are “middle-ground” KPS solutions as well. For instance, in random key predistribution schemes (e.g., [8, 9]), each node is preloaded with a random subset of keys from a key pool. To generate a pairwise key, two deployed nodes exchange the indices of their keys to discover whether or not they share any keys. If the two nodes share at least one key, they use the common key(s) to generate a pairwise key. These solutions are memory-friendly, and are resilient against node capture.

Similar to the random KPSs, the Blom scheme [10] (which is the focus of this work) preloads each node with a number of secret keys. It, however, uses a deterministic key generation scheme, and brings two main advantages over random KPSs. First, it enables any two nodes in the network to generate a pairwise key with probability 100%. Second, and perhaps more importantly, the Blom scheme provides the highest resilience against node capture in the sense that the network remains completely secure as long as the number of compromised nodes is less than the number of secret keys preloaded in each node. For instance, if each node is preloaded with 200 secret keys, the Blom scheme guarantees that an adversary who has captured and extracted all the secret keys of up to 199 nodes gains no information about the pairwise keys between uncaptured nodes. This fully discourages/defends against small-scale attacks, which is important as small-scale attacks are less expensive and harder to detect than large-scale attacks [9]. We also note that in the Blom scheme, when a new node is added to the network, the existing nodes do not need to receive any additional secret keys. As will be describe in Section 3, this is because two nodes only require to know their public information in order to establish a pairwise key [10].

To put the above advantages into perspective, consider for instance the *q*-composite key scheme, a random KPS designed to improve resiliency against small-scale attacks [9]. Suppose each node is preloaded with 200 keys, and that the probability that two nodes being able to generate a pairwise key is set to 50% (this is a parameter in the *q*-composite key scheme). Then, as demonstrated in [9], with probability of about 57% the communication between two uncaptured nodes is compromised if an adversary captures 150 nodes. The resilience of the *q*-composite key scheme becomes even worse if we increase the probability that two nodes are being able to generate a pairwise key. In the Blom scheme, on the other hand, any two nodes are able to generate a pairwise key with probability 100%. Moreover, all the communications between uncaptured nodes remain secure if an adversary capture 150 nodes.

A limiting factor of the Blom scheme for computationally constrained devices is that its pairwise key generation requires many field multiplications. To address this, in this work, we introduce TurboBlom, our fast variant of the Blom scheme that does not require any field multiplications, hence is significantly faster (e.g., about three orders of magnitude faster as shown in Section 4) than the Blom scheme.

Despite being significantly lighter than the Blom scheme, we analytically show that TurboBlom has nearly the same level of resilience against node capture as the original Blom scheme. In particular, we show that the gap between the resilience of TurboBlom and Blom can be made exponentially small with respect to the number of pre-loaded keys. Consequently, when the number of preloaded keys is large enough (e.g. larger than 30), TurboBlom provides practically the same level of resilience against node capture as the Blom scheme itself. In addition, in Section 4, we propose techniques based on the use of finite fields of Mersenne prime order to improve resilience of TurboBlom, which is particularly helpful when the number of preloaded keys is rather small.

Finally, we demonstrate how to use TurboBlom to provide efficient (in terms of memory and computation) and resilient (against node capture and Sybil attack) sender authentication in RPL [11]. RPL is the standard routing protocol for LLNs. These networks are a major component of IoT, and have a wide scope of applications including industrial monitoring and building automation. Consequently, the security of these networks is of great importance, especially when they are deployed in critical infrastructures such as smart grids [12].

In summary, we make the following contributions:

- We introduce TurboBlom, a fast variant of the Blom scheme, which is significantly lighter than the original Blom scheme, hence is more suitable for constrained devices. The lightness of Turboblom is specially appreciated when we note that a key exchange/distribution scheme with non-negligible computational requirement can expose devices to denial of service attacks.
- We analyze the resilience of TurboBlom against node capture and show that TurboBlom can offer practically the same level of resilience against node capture as the Blom scheme.
- We propose a new secure mode for RPL using TurboBlom to enable efficient sender authentication. This new mode is particularly useful as many emerging applications in RPL networks rely on point-to-point communications (as opposed to merely relying on communications with the gateway).

The rest of the paper is organized as follows. Section 2 overviews related work. Section 3 covers the basics of the Blom Scheme. We introduce TurboBlom in Section 4, and analyze its resilience against node capture. We propose TAM in Section 5, and conclude the paper in Section 7.

## 2 Related work

### Key predistribution

Early on, in the rise of large-volume industrial and consumer applications of wireless sensor networks, Carman, Kruus, and Matt [13] noticed that an adversary can physically capture nodes and acquire their data, since in practice they are typically left in an unattended/hostile environments and it is too costly, at large, to equip them with tamper-resistant hardware. In the quest for a solution, Eschenour and Gligor proposed the first random KPS in their seminal work [8]. Since then, many variants of this basic scheme have been proposed. These variants include schemes that use deterministic methods (usually based on combinatorial designs or error-correcting codes) in selecting subsets of keys for each node [14–20], and *multiple space* KPSs [21–23] which combine subset-based schemes with other key predistribution schemes such as the Blom scheme [10].

A notable variant of the Eschenour and Gligor (EG) scheme is the *q*-composite KPS due to Chan, Perrig and Song [9]. The *q*-composite scheme generalizes the EG scheme by requiring two devices to have at least *q* secret keys in common in order to establish a pairwise key. The *q*-composite offers greater resilience against node capture than EG when the number of nodes captured is relatively small, but is more vulnerable once a large number of nodes have been captured. This can be a desirable trade-off as small-scale attacks are less expensive and harder to detect [9].

When it comes to small-scale attacks, the optimum resilience is achieved by the Blom scheme [10]—it guarantees that the capture of a small number of nodes (up to the number of stored keys minus one) does not compromise any link between uncaptured nodes. In addition, the Blom scheme guarantees that any two nodes in the network are able to establish a pairwise key, whereas in the EG scheme and its variants, two nodes may not be able to establish a pairwise key as they may not have any secret key in common. The above advantages of the Blom scheme come at a price: to establish a pairwise key in the Blom scheme, a node must compute several field operations, including several computationally demanding field multiplications. In this work, we attempt to significantly reduce this cost by eliminating demanding field multiplications, while maintaining the scheme’s high resilience against node capture. Our method is based on using random generator matrices such as random zero-one matrices in the Blom scheme.

### RPL

Since RPL standardization in 2012, its security attracted a significant amount of research work on investigating potential threats, and proposing mitigation techniques for security attacks. In particular, many recent works have aimed at mitigating the sender’s authentication problem (or closely related problems such as the Sybil attack) in RPL networks.

For instance, in a recent work, Raoof *et al.* [24] proposed a new secure mode, Chained Secure Mode (CSM), for the RPL protocol using intra-flow network coding. In CSM, each node encodes its outgoing messages using a randomly generated code, and updates its neighbors with the next code. While CSM can protect the network against external attackers, it is vulnerable to internal attacks. It is because CSM assumes that the first message comes from the original sender. Therefore, an internal attacker can convince a node, say, *u* of being another node, say *v*, if the attacker sends the first message to *u* pretending to be *v*.

In another work, Airehrour *et al.* [25] proposed SecTrust-RPL, a new security framework for RPL. The authors proposed to install extra hardware on IoT devices to calculate a trust factor for each node. The trust factor is calculated based on successful packet transmissions between nodes. They showed that SecTrust-RPL can significantly mitigate the impact of Rank attack and Sybil attack. To protect the network against Sybil attack, the author proposed to bind the physical location of each node with its identity. Although this method can provide some degree of mitigation to the sender’s authentication problem, a node can still send messages using another node’s ID.

Despite many attempts in the literature (e.g., [26–29]) there is still no lightweight countermeasure that can prevent an adversary from spoofing, i.e. claiming the identity of a valid uncaptured node. In this work, however, we present a lightweight solution that prevents an adversary to use the identity of an uncaptured node unless the adversary extracts security keys of at least λ + 1 nodes, where λ is a security parameter.

## 3 Overview of the Blom scheme

In this section, we provide an overview of the Blom scheme. Table 1 provides the list of symbols used in this paper.

**Table 1:** List of symbols.

Symbol	Description
λ + 1	No. of secret keys stored in each node
*F*_*q*_	Finite field of order *q*
*F*_*p*_	Finite field of order *p*, where *p* is a prime
*G*	The generator matrix
*D*	The private matrix
*A*	The matrix obtained from *D* and *G* as *A* = (*DG*)^*T*^
*K*_*i*,*j*_	Pairwise key between nodes *i* and *j*
S	The set of elements of a confined generator matrix

### Bootstrapping phase

Consider a network with at most *n* nodes. In the “bootstrapping phase” of the Blom scheme, a trusted server generates two matrices: a public matrix *G* referred to as the *generator matrix*, and a private matrix *D*. The matrix *G* is a (λ + 1) × *n* matrix over *F*_*q*_, where *F*_*q*_ denotes the finite field of order *q*, and λ is a security parameter. The matrix *D*, on the other hand, is a (λ + 1)×(λ + 1) symmetric matrix over *F*_*q*_. The matrix *D* is a random matrix uniformly distributed over the set of all symmetric matrices over *F*_*q*_.

After generating matrices *D* and *G*, the server computes

$$\begin{array}{c} A={\left(DG\right)}^{T}, \end{array}$$ (1)

where (*DG*)^*T*^ denotes the transpose of *DG*. It then preloads node *u*_*i*_, 1 ≤ *i* ≤ *n*, with the *i*th row of matrix *A*, denoted *A*_*i*,:_, and the *i*th column of matrix *G*, denoted *G*_:,*i*_.

### Key establishment phase

Let *K* = *AG*. Since *D* is symmetric, we get

$$\begin{array}{c} K^{T}={\left(AG\right)}^{T}=G^{T}A^{T}\overset{\left(1\right)}{=}G^{T}\left(DG\right)=G^{T}D^{T}G\overset{\left(1\right)}{=}AG=K, \end{array}$$ (2)

thus *K* is a symmetric matrix, i.e., *K*_*i*,*j*_ = *K*_*j*,*i*_. In the Blom scheme, *K*_*i*,*j*_ (which is equal to *K*_*j*,*i*_) is used as the pairwise key between *u*_*i*_ and *u*_*j*_.

**Fig 1:**
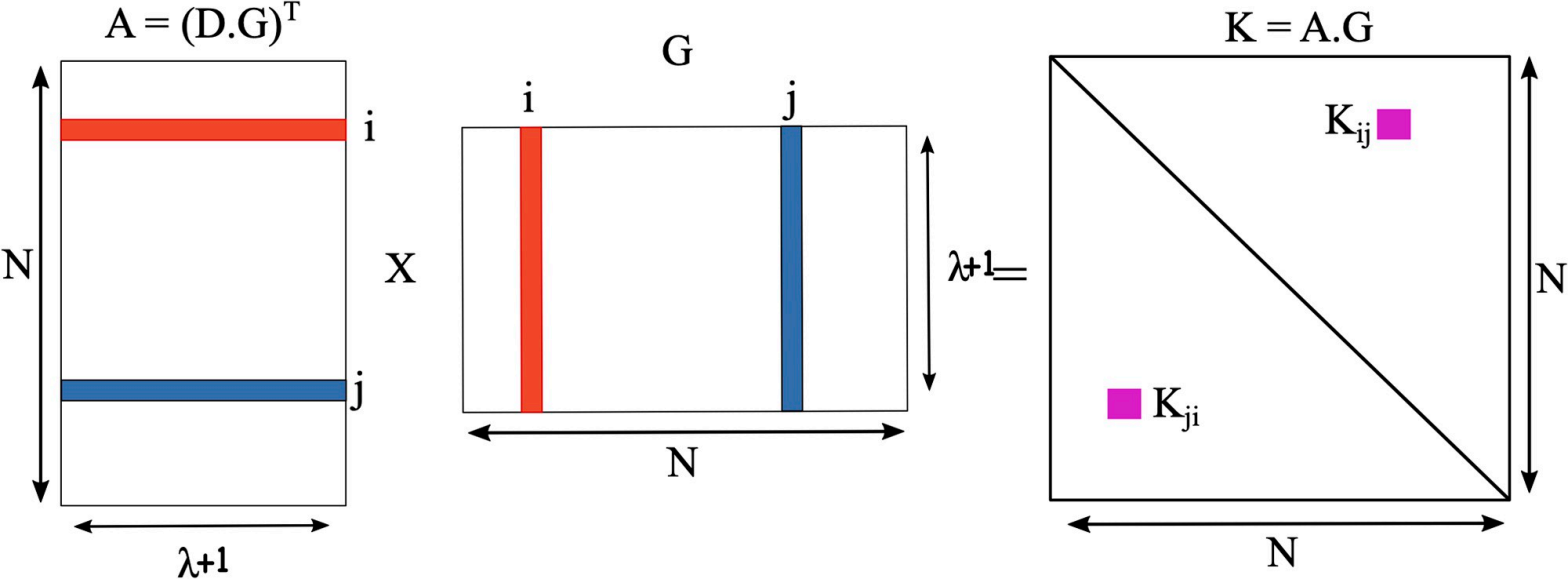
The pairwise key between nodes *u*_*i*_ and *u*_*j*_ is the element *K*_*i*,*j*_ of matrix *K*, where *K* = *A* ⋅ *G*.

To generate the pairwise key *K*_*i*,*j*_, in a so-called *key establishment phase*, nodes *u*_*i*_ and *u*_*j*_ first exchange their public information, i.e., their columns of *G*. Then, as illustrated in Fig 1, they generate their pairwise key as

$$\begin{array}{c} K_{i,j}=\left\langle A_{i,:},G_{:,j}\right\rangle =\left\langle A_{j,:},G_{:,i}\right\rangle \end{array}$$ (3)

where 〈***x***, ***y***〉 denotes the inner product of vectors ***x*** and ***y***.

### Resilience of the Blom scheme

If every λ + 1 columns of the generator matrix *G* are linearly independent, it can be proven that no information about the pairwise keys between uncaptured nodes is revealed if the number of captured nodes is at most λ [10, 22]. We will generalize this result later in Theorem 2.

### Constructing matrix G

When *q* > *n*, we can construct a matrix *G* in which every λ + 1 columns are linearly independent. For instance, it can be shown that when *q* > *n*, every λ + 1 columns of the following Vandermonde matrix (with *g* ∈ *F*_*q*_ being a primitive element) are linearly independent [22].

$$\begin{array}{c} G=[\begin{array}{ccccc} 1 & 1 & 1 & \cdots & 1\\ g & g^{2} & g^{3} & \cdots & g^{n}\\ g^{2} & {\left(g^{2}\right)}^{2} & {\left(g^{3}\right)}^{2} & \cdots & {\left(g^{n}\right)}^{2}\\ & & & \vdots & \\ g^{\lambda } & {\left(g^{2}\right)}^{\lambda } & {\left(g^{3}\right)}^{\lambda } & \cdots & {\left(g^{n}\right)}^{\lambda } \end{array}] \end{array}$$ (4)

An advantage of using a Vandermonde matrix as the generator matrix in the Blom scheme is that node *u*_*i*_ only needs to store *g*^*i*^, instead of the entire *i*th column of *G* (i.e. *G*_:,*i*_), since the entire column *G*_:,*i*_ can be constructed from *g*^*i*^—to construct the *i*th column of *G*, however, the node needs to perform λ − 1 field multiplications to compute (*g*^*i*^)^2^, (*g*^*i*^)^3^, …, (*g*^*i*^)^λ^ from *g*^*i*^.

**Example 1**. *Consider a network with* *n* = 5 *nodes. Let* *q* = 19, λ = 2, *and*

$$G=[\begin{array}{ccccc} 1 & 1 & 1 & 1 & 1\\ 2 & 4 & 8 & 16 & 13\\ 4 & 16 & 7 & 9 & 17 \end{array}]\;D=[\begin{array}{ccc} 6 & 15 & 14\\ 15 & 1 & 8\\ 14 & 8 & 3 \end{array}]$$

*Notice that Matrix D is symmetric, and every four columns of matrix G are linearly independent. The matrix A is then calculated as:*

$$A={\left(DG\right)}^{T}=[\begin{array}{ccc} 16 & 11 & 4\\ 5 & 14 & 18\\ 15 & 3 & 4\\ 11 & 8 & 17\\ 2 & 12 & 17 \end{array}]$$

*Before being deployed in the network, node* *u*_*i*_ *is preloaded with the* *i*th *column of* *G* *and the* *i* *th row of* *A* *as its public and private information, respectively. Assume nodes* *u*_2_ *and* *u*_5_ *want to communicate securely with each other after they are deployed in the network. To establish a pairwise key, node* *u*_2_ *calculates the following*:

$$K_{2,5}=\left\langle A_{2,:},G_{:,5}\right\rangle =[\begin{array}{ccc} 5 & 14 & 18 \end{array}]\cdot [\begin{array}{c} 1\\ 13\\ 17 \end{array}]=18$$

*Similarly, node* *u*_5_ *computes* *K*_5,2_ *as*

$$K_{5,2}=\left\langle A_{5,:},G_{:,2}\right\rangle =[\begin{array}{ccc} 2 & 12 & 17 \end{array}]\cdot [\begin{array}{c} 1\\ 4\\ 16 \end{array}]=18$$

*In this example, the secret pairwise key between nodes* *u*_2_ *and* *u*_5_ *is 18. Note that, to provide enough security, the order of finite fields used in practice will be much larger than 19*.

## 4 TurboBlom

In this section, we introduce TurboBlom, and show how it significantly reduces the computational cost of the Blom scheme while maintaining a high resilience against node capture.

**Fig 2:**
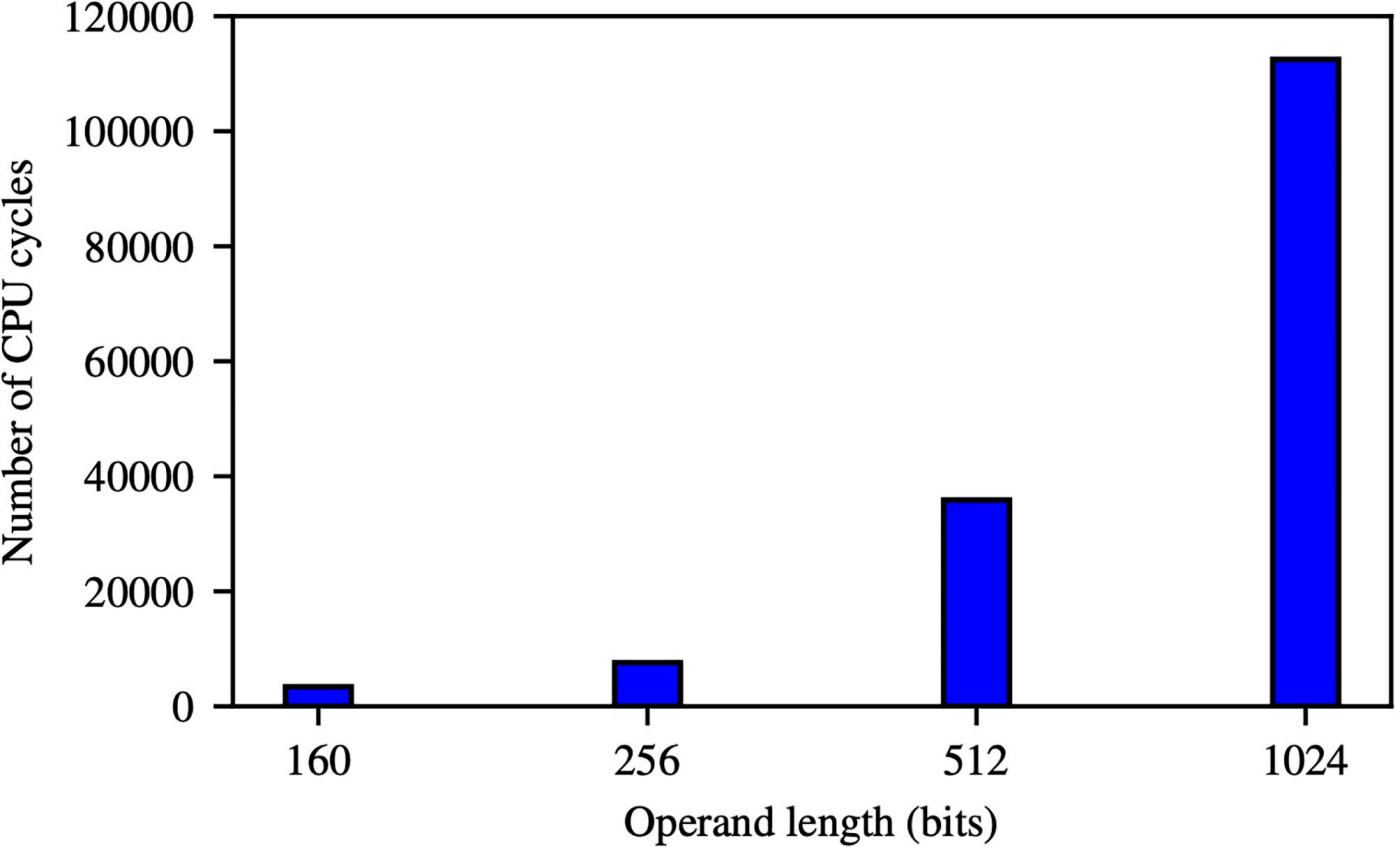
The average number of CPU cycles needed to compute a single field multiplication in MSP430 processor.

**Fig 3:**
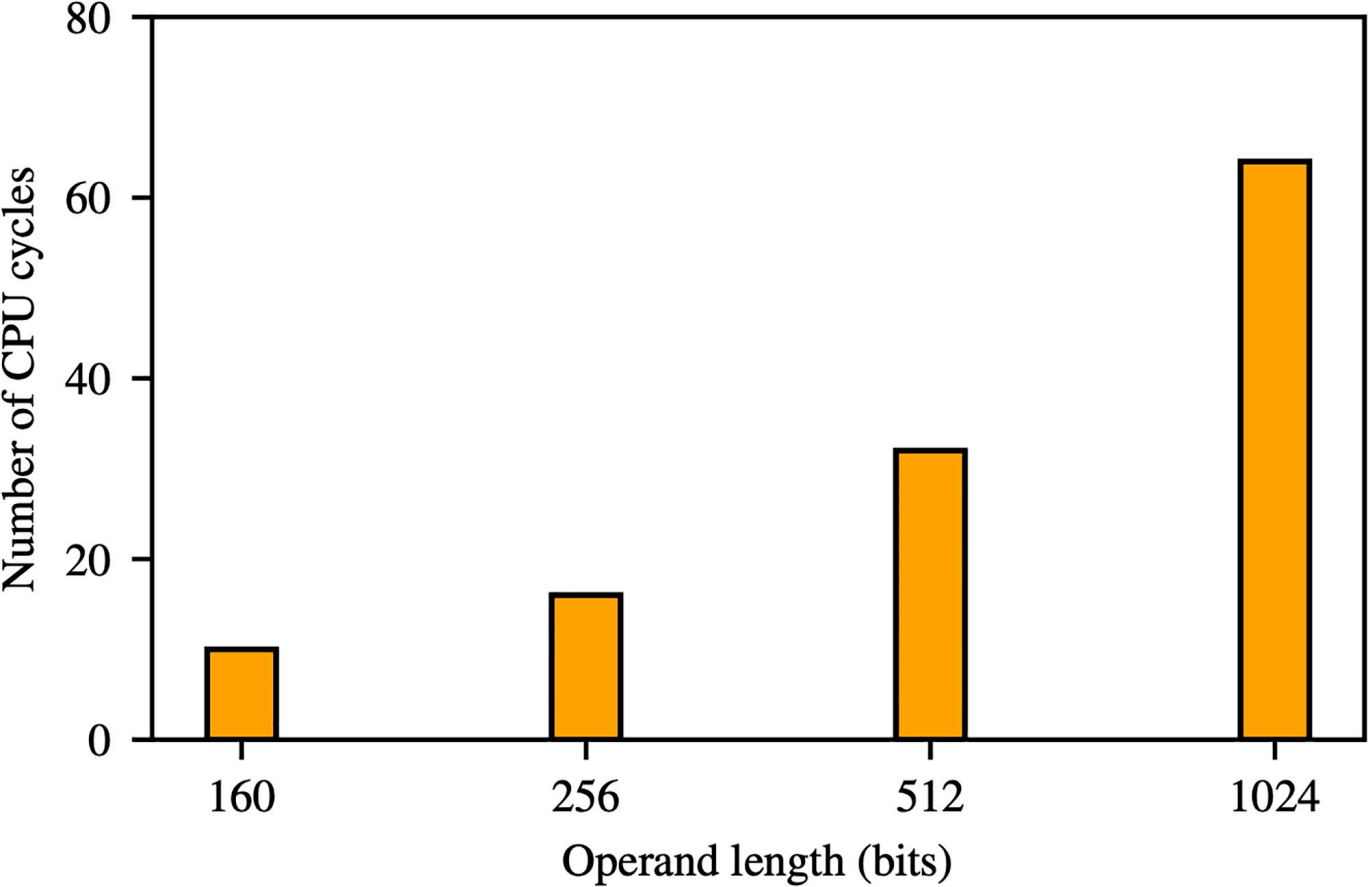
The average number of CPU cycles needed to compute a single field addition in MSP430 processor.

Recall that nodes *u*_*i*_ and *u*_*j*_ need to compute the inner product (3) to generate a pairwise secret key. Computing this inner product requires λ + 1 field multiplications and λ field additions. Computing a field multiplication, in general, is considerably harder than computing a field addition. For example, Figs 2 and 3 show the average number of CPU clock cycles needed to compute a field multiplication and a field addition, respectively, on 16-bit MSP430 microprocessor family—this is a family of processors commonly used in IoT devices such as Tmote Sky and Zolertia Z1 [30, 31]. As shown in these figures, computing a field multiplication over a field of order 256 bits, for instance, requires 547 times more clock cycles than computing a field addition. Since computing field multiplications is the main cost of generating a pairwise key in the Blom scheme, in TurboBlom we aim to eliminate field multiplications in order to speed up pairwise key generation.

### Eliminating field multiplications

As mentioned earlier, a node must compute the inner product (3) to generate a pairwise key. We can eliminate the need for field multiplication in (3) if we use a “confined” generator matrix *G*. In a confined matrix, elements can only come from a subset $S\subset F_{q}$. For example, if we set S={0,1}, we limit the elements of the generator matrix *G* to 0 and 1. That is, each element of *G* can be either the number 0 or the number 1, where 0 and 1 represent, respectively, the additive and multiplicative identities of *F*_*q*_.

Such a “zero-one matrix” (We refer to the matrix as zero-one matrix rather than binary matrix to stress that the matrix is over *F*_*q*_ rather than *F*_2_, although its elements are limited to zero and one) eliminates the need for field multiplication, because it turns every multiplications in (3) into a multiplication by 0 or a multiplication by 1 (which are both trivial). This makes pairwise key generation in the Blom scheme significantly faster. The important concern with using a confined generator matrix, such as a zero-one generator matrix, is its potential negative impact on the resilience of the scheme. A major contribution of this work is to answer this concern. Looking ahead, we later in this section show that this impact can be made exponentially small in practice.

**Remark 1**. *Although the elements of* *G* *are limited to a small subset of* *F*_*q*_, *all the operations in computing the inner product* (3) *are still performed in the field* *F*_*q*_. *Moreover, the pairwise key between nodes can still take any value from* *F*_*q*_, *because the elements of matrix* *D* *can take any value from* *F*_*q*_ (*see Example* 2). *We will formally prove this in Theorem* 2.

We can also limit the elements of matrix *G* to

S={-1,0,1}.

This, too, eliminates field multiplications, because multiplication by −1 is a fast operation (recall that −1 ≠ 1 in any finite field of odd order). This idea can be extended to even larger sets S if we use a finite field *F*_*p*_ in which *p* is a Mersenne prime (in general, it is possible to use a field with characteristic *p*, where *p* is a Mersenne prime). A Mersenne prime is a prime of the form 2^*n*^ − 1, where *n* is an integer. An interesting property of *F*_*p*_, with *p* being a Mersenne prime, is that multiplying a number *a* ∈ *F*_*p*_ by a power of two (i.e, a number of the form 2^i^) is simply a circular shift of *a*, when *a* is represented in binary. In addition, negating a number *a* (i.e., computing −*a*) can be performed easily by reversing all the bits of *a*, when *a* is represented in binary. Considering the above properties, we can set

$$\begin{array}{c} S=\{-2^{n-1},\ldots ,-2^{1},-2^{0},0,2^{0},2^{1},\ldots ,2^{n-1}\}. \end{array}$$ (5)

Since all the non-zero elements of the above set S are powers of two, every field multiplication is either eliminated (due to multiplication by zero), or replaced by a simple shift operation (due to multiplication by a power of two), possibly followed by bit reversal (due to negation).

**Remark 2**. *The set* S *defined in* (5) *has* 2*n* + 1 *distinct* *elements, i.e*. |S|=2n+1. *This is because* $\left(2^{i}\neq 2^{j}\;\text{mod}\;p\right)$ *and* $\left(2^{i}\neq -2^{j}\;\text{mod}\;p\right)$ *for any* 0 ≤ *i* ≠ *j* ≤ *n* − 1, *where* *p* = 2^*n*^ − 1.

**Example 2**. *Consider a network with* *n* = 4 *nodes*, *u*_1_, *u*_2_, *u*_3_ *and* *u*_4_. *Let* λ = 1, *and*

$$G=[\begin{array}{cccc} 0 & 1 & 1 & 1\\ 1 & 0 & 1 & -1 \end{array}]\;D=[\begin{array}{cc} a & c\\ c & b \end{array}]$$

*where the elements of the secret matrix* *D* (*i.e.*, *a*, *b*, *and* *c*) *are random numbers uniformly distributed over* *F*_*q*_, *and* *q* *is an odd number (hence* −1 ≠ 1). *Notice that Matrix* *D* *is symmetric, and every two columns of* *G* *are linearly independent. The matrix* *A* *is then calculated as*:

$$A={\left(DG\right)}^{T}=[\begin{array}{cc} c & b\\ a & c\\ a+c & c+b\\ a-c & c-b \end{array}]$$

**Fig 4:**
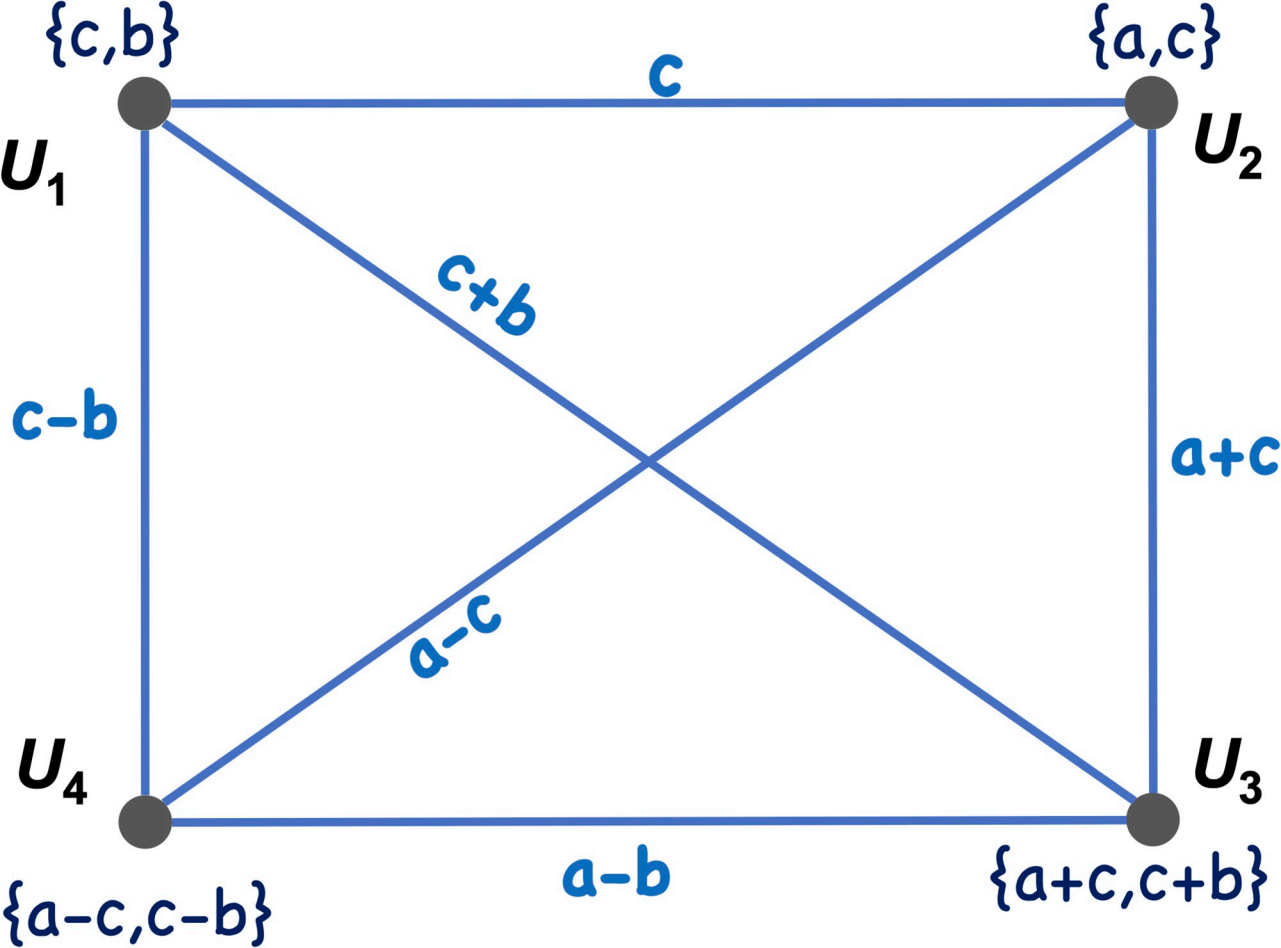
Each node *u*_1_, *u*_2_, *u*_3_, and *u*_4_ is preloaded with a pair of numbers. The number on each edge is the pairwise key between the two nodes incident to the edge.

*Before being deployed in the network, node* *u*_*i*_ *is preloaded with the* *ith row of* *A*. *For instance*, *u*_1_ *is preloaded with secret numbers* *c* *and* *b*, *and* *u*_2_ *is preloaded with secret numbers* *a and c*. Fig 4 *shows the set of two secret numbers each node is preloaded with, as well as the pairwise key between each pair of nodes (the number on each edge). For instance, as shown in the figure, the pairwise key between* *u*_2_ *and* *u*_3_ *is the number* *a* + *c*. *Note that, although the elements of matrix* *G* *are limited to* S={-1,0,1}, *the pairwise key between two nodes can take any number from* *F*_*q*_. *This is because the elements of* *D* (i.e., *a*, *b*, *and* *c*) *are uniformly distributed over* *F*_*q*_. *Also, notice that each node can compute a pairwise key with any other node using its preloaded numbers. For instance*, *u*_4_ *can compute the pairwise key* *a* − *b* *with node* *u*_3_ *by adding its two secret numbers, i.e.* (*a* − *c*) + (*c* − *b*).

**Fig 5:**
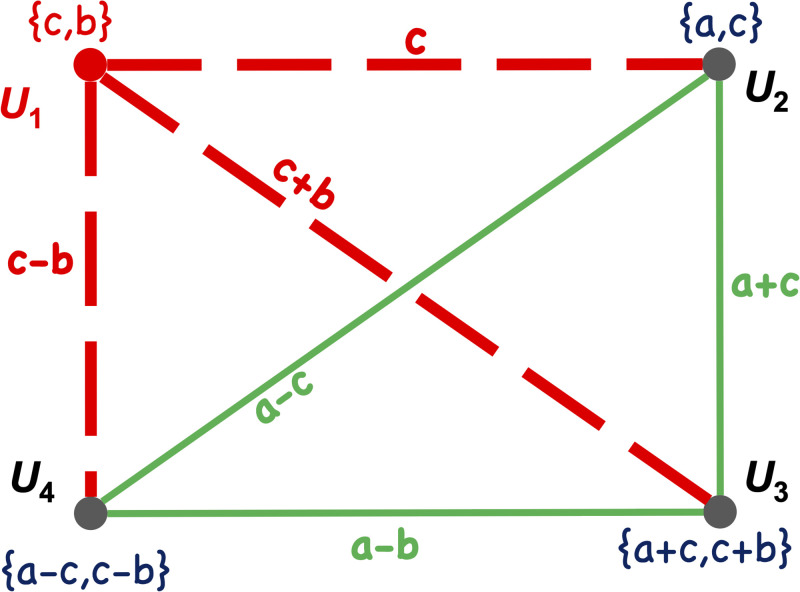
The adversary captures node *u*_1_ and extracts the secret numbers *c* and *b*. This will compromise the links between *u*_1_ and the other three nodes (the red dashed links). The adversary, however, gains no information about the pairwise keys between *u*_2_, *u*_3_ and *u*_4_, which are *a* − *c*, *a* − *b* and *a* + *c*. This is because *a* is uniformly distributed over *F*_*q*_, hence *a* − *c*, *a* − *b* and *a* + *c* are all uniformly distributed over *F*_*q*_.

*As illustrated in* Fig 5, *suppose that the adversary captures node* *u*_1_ *and extracts the secret numbers* *c and b*. *This will clearly compromise the links between node* *u*_1_ *and the remaining three nodes* *u*_2_, *u*_3_ *and* *u*_4_. *The adversary, however, gains no information about pairwise keys between uncaptured nodes (i.e., pairwise keys corresponding to green solid links) because the adversary has no information about* *a* (*as* *a* *is uniformly distributed over* *F*_*q*_). *Similarly, we can verify that if adversary captures another node, they do not gain any information about the pairwise key between uncaptured nodes*.

**Remark 3**. *In Example 2, the elements of matrix* *G* *were limited to the set* S={-1,0,1}. *Yet, the pairwise keys between two nodes are uniformly distributed over* *F*_*q*_. *Furthermore, the adversary gains no information about the pairwise key of two nodes by capturing another node in the network*.

### Speedup of TurboBlom

In this section, we compare the speed of the TurboBlom and Blom schemes. In the next section, we will compare the resilience of the two schemes, a considerably more challenging task.

To compare the speed of the two schemes, we can compare the number of operations that each scheme needs. This is not difficult as both schemes perform the same inner inner product (3) to generate pairwise key. In the Blom scheme, computing this inner product needs λ + 1 field multiplications and λ filed additions. In the TurboBlom scheme, however, no field multiplication is required: any field multiplication in the Blom scheme is either completely eliminated in the TurboBlom scheme or is replaced with a simple operation such as circular shift, which is essentially as fast as field addition. Using this insight, the following theorem evaluates the speedup of TurboBlom over the Blom scheme.

**Theorem 1**. *Suppose field multiplication and addition require, respectively*, *m and a* *units of time*.

*Then, the TurboBlom’s speedup over the Blom scheme is at least*

$$\frac{1}{2}\cdot (\frac{m}{a}+1).$$

*Moreover, if we use a zero-one matrix in TurboBlom, the above speedup is improved to at least* $(\frac{m}{a}+1),$ *in the worst case, and to at least* $2\cdot (\frac{m}{a}+1),$ *in the average*.

*Proof*. To generate a pairwise key specified by (3), the Blom scheme requires λ + 1 field multiplications and λ filed additions. This translates to the the computation time of

(λ+1)·m+λ·a

In TurboBlom, each field multiplication is either eliminated or replaced with a simple operation (circular shift or bit reverse) which is practically as fast as field addition. Therefore, in the worst case, TurboBlom needs 2λ + 1 field additions, which translates to the computation time of

(λ+1)·a+λ·a.

Therefore, we get the speedup of at least

$$\frac{(\lambda +1)\cdot m+\lambda \cdot a}{(\lambda +1)\cdot a+\lambda \cdot a}\geq \frac{1}{2}\cdot (\frac{m}{a}+1).$$

If TurboBlom uses a zero-one matrix, then all of the field multiplications are eliminated. Therefore, in this case the speedup will be

$$\frac{(\lambda +1)\cdot m+\lambda \cdot a}{0\cdot m+\lambda \cdot a}\geq \frac{m}{a}+1.$$

We note that, on average, half of all of the field additions are eliminated (due to zeros in the generator matrix) when the elements of the zero-one generator matrix are selected uniformly at random. Let 1 ≤ λ^′^ ≤ λ be a random variable denoting the number of required field additions. Then, the expected speedup can be calculated as

$$\begin{array}{cc} E[\frac{(\lambda +1)\cdot m+\lambda \cdot a}{0\cdot m+\lambda^{\prime }\cdot a}] & =E[\frac{(\lambda +1)\cdot m+\lambda \cdot a}{\lambda^{\prime }\cdot a}]\\ & =(\frac{(\lambda +1)\cdot m+\lambda \cdot a}{a})\cdot E[\frac{1}{\lambda^{\prime }}]\\ & \geq (\frac{m}{a}+1)\cdot \lambda \cdot E[\frac{1}{\lambda^{\prime }}]\\ & \geq (\frac{m}{a}+1)\cdot \lambda \cdot \frac{1}{E[\lambda^{\prime }]}\\ & =(\frac{m}{a}+1)\cdot \lambda \cdot \frac{1}{\left(\frac{\lambda }{2}\right)}\\ & =2\cdot (\frac{m}{a}+1), \end{array}$$

Where the last inequality is by the fact that

$$E\left[\frac{1}{x}\right]\geq \frac{1}{E[x]}$$

for any positive random variable *x*. Note that Jensen’s inequality states that E[ϕ(x)]≥ϕ(E[x]) for any convex function *ϕ*. The function $\phi \left(x\right)=\frac{1}{x}$ is convex when *x* > 0, hence the inequality.

**Example 3**. *The speedup of TurboBlom can be significant in practice. For instance, as illustrated in* Figs 2 and 3, *when the field size is 256 bits, we have* $\frac{m}{a}\approx 547$. *Therefore, if we use a zero-one matrix in TurboBlom, we can expect a speedup of around* 2(547 + 1) = 1, 096 *over the Blom scheme (i.e., TurboBlom requires about three orders of magnitude less number clock cycles as the Blom scheme to compute a pairwise key*).

### Resilience of TurboBlom

As mentioned earlier, we can eliminate all the field multiplications in the pairwise key generation process by using a confined generator matrix *G*. This makes TurboBlom significantly lighter and faster than the original Blom scheme. In addition, as we will show in the remaining of this section, TurboBlom can maintain a high resilience against node capture. To this end, we present Theorem 2, which proves that an adversary who has captured a number of nodes and extracted their secret keys gains no information about the pairwise key between any two uncaptured nodes *u*_*i*_ and *u*_*j*_ if neither the *i*th column nor the *j*th column of *G* is a linear combination of the columns of *G* that correspond to the captured nodes. Notably, this result holds for any generator matrix *G* including zero-one matrices.

Prior to presenting Theorem 2, let us first formally define what we mean by “adversary gains no information”.

**Definition 1** (Adversary gaining no information). *Let us fix the generator matrix* *G* *to an arbitrary* (λ + 1) × *n* *matrix over* *F*_*q*_. *Suppose the adversary has captured and extracted secret keys of nodes* *u*_*i*_, i∈L, *where* L *represents the set of captured nodes. Let the* 1 × (λ + 1) *vector* *A*_*i*_ *over* *F*_*q*_ *denote the secret keys of node* *u*_*i*_, i∈L. *Let* D *be the set of all* (λ + 1) × (λ + 1) *symmetric matrices that, when used with the generator matrix* *G*, *result in* *A*_*i*_ *for every* i∈L, *that is*

$$D=\{D|\forall i\in L:{\left(D\cdot G_{:,i}\right)}^{T}=A_{i}\}$$

*Given the above setup, consider a probabilistic experiment in which the random variable* *D* *is uniformly distributed over* D . *Let* *K*_*s*,*d*_ *be the random variable representing the pairwise key between nodes* *u*_*s*_ *and* *u*_*d*_. *We say that the* *adversary gains no information* *about* *K*_*s*,*d*_ *if and only if* *K*_*s*,*d*_ *is uniformly distributed over* *F*_*q*_.

**Lemma 1**. *Let* V *be a subspace of the vector space* $F_{q}^{m}$, *where* *m* *is a positive integer. Let* W *denote the null space of* V.

*Then, for every vectors* $y_{1},y_{2}\in F_{q}^{m}\backslash V$ , *there exists a vector* w∈W *such that* ***w*** *is not orthogonal to either* ***y***_1_ *or* ***y***_2_, *i.e*.

$$\forall y_{1},y_{2}\in F_{q}^{m}\backslash V,\;\exists w\in W:\;\left\langle y_{1},w\right\rangle \neq 0\;\wedge \;\left\langle y_{2},w\right\rangle \neq 0.$$

*Proof*. A vector $v\in F_{q}^{m}$ is orthogonal to every vector in W if and only if v∈V. Since $y_{1}\notin V$, then there must exist a vector in W which is not orthogonal to ***y***_1_. Therefore, the set $W_{1}$ defined as

$$W_{1}=\left\{w\in W|\left\langle y_{1},w\right\rangle \neq 0\right\}$$

is non-empty. Similarly, the set $W_{2}$

$$W_{2}=\left\{w\in W|\left\langle y_{2},w\right\rangle \neq 0\right\}$$

must be non-empty. If $(W_{1}\cap W_{2})\neq \emptyset$, then we are done because any vector

$$w\in W_{1}\cap W_{2}$$

is not orthogonal to either ***y***_1_ or ***y***_2_. Otherwise, we can set ***w*** = ***w***_1_ + ***w***_2_, where $w_{1}\in W_{1}$ and $w_{2}\in W_{2}$. Note that w∈W. Furthermore, $w_{1}\notin W_{2}$ and $w_{2}\notin W_{1}$ because $(W_{1}\cap W_{2})=\emptyset$. Therefore, by the definition of sets $W_{1}$ and $W_{2}$, we get

$$\begin{array}{c} \langle y_{1},w_{2}\rangle =0 \end{array}$$ (6)

and

$$\begin{array}{c} \langle y_{2},w_{1}\rangle =0. \end{array}$$ (7)

Thus

$$\begin{array}{cc} \left\langle y_{1},w\right\rangle & =\langle y_{1},\left(w_{1}+w_{2}\right)\rangle \\ & =\left\langle y_{1},w_{1}\right\rangle +\left\langle y_{1},w_{2}\right\rangle \\ & \overset{\left(6\right)}{=}\left\langle y_{1},w_{1}\right\rangle +0\\ & \neq 0. \end{array}$$

and

$$\begin{array}{cc} \left\langle y_{2},w\right\rangle & =\langle y_{2},\left(w_{1}+w_{2}\right)\rangle \\ & =\left\langle y_{2},w_{1}\right\rangle +\left\langle y_{2},w_{2}\right\rangle \\ & \overset{\left(7\right)}{=}0+\left\langle y_{2},w_{2}\right\rangle \\ & \neq 0. \end{array}$$

Thus, the vector ***w*** = ***w***_1_ + ***w***_2_ is not orthogonal to either ***y***_1_ or ***y***_2_.

**Lemma 2**. *Let* *B* *be a non-zero matrix over* *F*_*q*_, *and* A *be a non-empty set of matrices over* *F*_*q*_ *such that*

$$\forall c\in F_{q}:\;A\in A\Rightarrow (A+c\cdot B)\in A.$$

*Then, the binary relation ∼ on* A *defined as*

$$\forall A_{1},A_{2}\in A:\;A_{1}∼A_{2}\Leftrightarrow A_{1}-A_{2}=c\cdot B\;\text{for}\;\text{some}\;c\in F_{q}$$

*is an equivalence relation, which partitions* A *into equivalent classes* [*A*_*i*_] *of size* *q*, *where* $A_{i}\in A$ *and*

$$\left[A_{i}\right]=\left\{A|A=A_{i}+c\cdot B,\;c\in F_{q}\right\}.$$

*Proof*. The binary relation ∼ on A is an equivalence relation because it is reflexive, symmetric and transitive:

- (Reflexivity) ∀A∈A:A∼A, because *A* − *A* = 0 ⋅ *B*, *c* = 0 ∈ *F*_*q*_.
- (Symmetry) $\forall A_{1},A_{2}\in A:$ (*A*_1_ ∼ *A*_2_) ⇒ (*A*_2_ ∼ *A*_1_), because
  $$\begin{array}{cc} \left(A_{1}∼A_{2}\right) & \Rightarrow A_{1}-A_{2}=c\cdot B,\;c\in F_{q}\\ & \Rightarrow A_{2}-A_{1}=\left(-c\right)\cdot B,\;-c\in F_{q}\\ & \Rightarrow (A_{2}∼A_{1}). \end{array}$$
- (Transitivity) $\forall A_{1},A_{2},A_{3}\in A:$
  $$\left(A_{1}∼A_{2}\right)\wedge \left(A_{2}∼A_{3}\right)\Rightarrow \left(A_{1}∼A_{3}\right)$$
  because
  $$\begin{array}{cc} & \left(A_{1}∼A_{2}\right)\wedge \left(A_{2}∼A_{3}\right)\\ & \Rightarrow \left(A_{1}-A_{2}=c_{1}\cdot B\right)\wedge \left(A_{2}-A_{3}=c_{2}\cdot B\right),\;c_{1},c_{2}\in F_{q}\\ & \Rightarrow A_{1}-A_{3}=\left(c_{1}+c_{2}\right)\cdot B,\;\left(c_{1}+c_{2}\right)\in F_{q}\\ & \Rightarrow (A_{1}∼A_{3}). \end{array}$$

Therefore, the binary relation ∼ partitions A into disjoint equivalence classes

$$\begin{array}{cc} \left[A_{i}\right] & =\{A|A∼A_{i}\}\\ & =\{A|A=A_{i}+c\cdot B,\;c\in F_{q}\}. \end{array}$$

Finally, note that the coefficient *c* ∈ *F*_*q*_ takes *q* distinct values. Furthermore, *B* is non-zero, thus

$$(A_{i}+c_{1}\cdot B=A_{i}+c_{2}\cdot B)\Leftrightarrow c_{1}=c_{2}.$$

Therefore, every class [*A*_*i*_] has exactly *q* distinct elements.

**Theorem 2**. *Let* *G* *be an arbitrary* (λ + 1) × *n* *matrix over* *F*_*q*_, *where* λ *and* *n* *are positive integers. Note that, as a special case*, *G* *can be a zero-one matrix. Suppose we use* *G* *as the generator matrix of the Blom scheme. Suppose the secret random matrix* *D* *is uniformly distributed over the set of all* (λ + 1) × (λ + 1) *symmetric matrices. Assume that an adversary has captured and extracted the secret keys of a set* L *of* *l* ≥ 1 nodes. *Let* ***g***_1_, ***g***_2_, …, ***g***_*l*_ *be the column vectors of* *G* *that correspond to these* *l* *captured nodes. Let* *u*_*s*_ *and* *u*_*d*_ *be any two uncaptured nodes, and* ***g***_*s*_ *and* ***g***_*d*_ *be the two column vectors of* *G* *that correspond to* *u*_*s*_ *and* *u*_*d*_, *respectively*.

*Then, the adversary gains no information about the pairwise key between* *u*_*s*_ *and* *u*_*d*_ *if and only if neither* ***g***_*s*_ *nor* ***g***_*d*_ *is a linear combination of* ***g***_1_, ***g***_2_, …, ***g***_*l*_.

*Proof*. Let ***a***_1_, ***a***_2_, …, ***a***_*l*_ be the row vectors of *A* that correspond to the captured nodes. We have

$$\begin{array}{c} \forall 1\leq i\leq l:\;a_{i}={\left(Dg_{i}\right)}^{T}, \end{array}$$ (8)

because *A* = (*DG*)^*T*^.

Suppose ***g***_*s*_ is a linear combination of ***g***_1_, ***g***_2_, …, ***g***_*l*_, that is

$$\begin{array}{c} g_{s}=\sum_{i=1}^{l}c_{i}\cdot g_{i}, \end{array}$$ (9)

for some *c*_*i*_ ∈ *F*_*q*_. The adversary knows the values of *c*_*i*_, 1 ≤ *i* ≤ *l*, because the generator matrix *G* is public. In addition, the adversary knows ***a***_*i*_, 1 ≤ *i* ≤ *l*, which are the secret keys of the captured nodes. Therefore, by (8) and (9), the adversary can compute ***a***_*s*_ as

$$\begin{array}{c} a_{s}=\sum_{i=1}^{l}c_{i}\cdot a_{i}, \end{array}$$ (10)

because

$$\begin{array}{cc} a_{s} & ={\left(Dg_{s}\right)}^{T}\\ & \overset{\left(9\right)}{=}{\left(D\cdot \sum_{i=1}^{l}c_{i}\cdot g_{i}\right)}^{T}\\ & ={\left(\sum_{i=1}^{l}c_{i}\cdot Dg_{i}\right)}^{T}\\ & =\sum_{i=1}^{l}c_{i}\cdot {\left(Dg_{i}\right)}^{T}\\ & \overset{\left(8\right)}{=}\sum_{i=1}^{l}c_{i}\cdot a_{i}. \end{array}$$

Therefore, if ***g***_*s*_ is a linear combination of ***g***_1_, ***g***_2_, …, ***g***_*l*_, the adversary can compute the secret keys of node *u*_*s*_ (i.e., ***a***_*s*_), thus can compute the pairwise key between *u*_*s*_ and any other node (including *u*_*d*_). Similarly, if ***g***_*d*_ is a linear combination of ***g***_1_, ***g***_2_, …, ***g***_*l*_, the adversary can compute the secret keys of node *u*_*d*_.

Next, we prove the challenging part, which is that the adversary gains no information about the pairwise key between *u*_*s*_ and *u*_*d*_ if neither ***g***_*s*_ nor ***g***_*d*_ is a linear combination of ***g***_1_, ***g***_2_, …, ***g***_*l*_. To this end, we assume that neither ***g***_*s*_ nor ***g***_*d*_ is a linear combination of ***g***_1_, ***g***_2_, …, ***g***_*l*_.

Let *G*_0_ be the submatrix of *G* that includes only the column vectors ***g***_*i*_, 1 ≤ *i* ≤ *l*. Let *A*_0_ be the submatrix of *A* that includes only the row vectors ***a***_*i*_, 1 ≤ *i* ≤ *l*. By (1), we have

$$A_{0}={\left(DG_{0}\right)}^{T}$$

or equivalently

$$\begin{array}{c} A_{0}^{T}=DG_{0} \end{array}$$ (11)

As in Definition 1, we define D to be the set of all symmetric matrices *D* that satisfy Eq (11). Note that the set D is not empty, because the secret matrix *D* used in the Blom scheme satisfies (11).

Let V denote the column space of *G*_0_, and W be the null space of V. Recall that neither ***g***_*s*_ nor ***g***_*d*_ is a linear combination of ***g***_1_, ***g***_2_, …, ***g***_*l*_. Thus, $g_{s},g_{d}\notin V$. Therefore, by Lemma 1, there exits w∈W such that ***w*** is not orthogonal to either ***g***_*s*_ or ***g***_*d*_. Let

$$w=(w_{1},w_{2},\ldots ,w_{\lambda +1})$$

and *B* be a (λ + 1) × (λ + 1) matrix over *F*_*q*_ whose *i*th row, 1 ≤ *i* ≤ λ + 1, is *w*_*i*_ ⋅ ***w***. The matrix *B* is symmetric because

$$B_{i,j}=B_{j,i}=w_{i}\cdot w_{j}.$$

Furthermore, we have

$$BG_{0}=0,$$

because each row of *B* is in W, hence is orthogonal to every column of *G*_0_. Therefore, we get

$$\forall c\in F_{q}:\;D\in D\Rightarrow \left(D+c\cdot B\right)\in D,$$

because

$$\begin{array}{cc} \left(D+c\cdot B\right)G_{0} & =DG_{0}+c\cdot BG_{0}\\ & =A_{0}^{T} \end{array}$$

that is (*D* + *c* ⋅ *B*) satisfies (11), hence is in D by definition. Let us define the binary relation ∼ on D as

$$\forall D_{1},D_{2}\in D:\;D_{1}∼D_{2}\Leftrightarrow D_{1}-D_{2}=c\cdot B\;\text{for}\;\text{some}\;c\in F_{q}.$$

By Lemma 2, the binary relation ∼ is an equivalence relation which partitions D into equivalent classes of size *q*. Let [*D*_*i*_] denote the *i*th, 1 ≤ *i* ≤ *k*, equivalent class represented by the matrix $D_{i}\in D$. Since the matrix *D* in the Blom scheme is uniformly distributed over D, it can be represented as

$$\begin{array}{c} D=D_{j}+r\cdot B, \end{array}$$ (12)

where *j* and *r* are random variables uniformly distributed over {1, 2, …, *k*}, and *F*_*q*_, respectively. The random variable *j* indicates the class to which matrix *D* belongs, and the random variable *r* indicates which element of the class [*D*_*j*_] is the matrix *D*.

Let

$$\begin{array}{c} a_{s}=\left\langle g_{s},w\right\rangle , \end{array}$$ (13)

and

$$\begin{array}{c} a_{d}=\left\langle g_{d},w\right\rangle , \end{array}$$ (14)

Note that *a*_*s*_ ≠ 0 and *a*_*d*_ ≠ 0, because ***w*** is not orthogonal to either ***g***_*s*_ or ***g***_*d*_. By (1) and (3), the pairwise key between *u*_*s*_ and *u*_*d*_ is

$$\begin{array}{cc} K_{s,d} & \overset{\left(3\right)}{=}a_{s}\cdot g_{d}\\ & \overset{\left(1\right)}{=}(g_{s}^{T}\cdot D)\cdot g_{d}\\ & \overset{\left(12\right)}{=}(g_{s}^{T}\cdot (D_{j}+r\cdot B))\cdot g_{d}\\ & =(g_{s}^{T}\cdot D_{j}+r\cdot (g_{s}^{T}\cdot B))\cdot g_{d}\\ & =(g_{s}^{T}\cdot D_{j}+r\cdot {\left(B\cdot g_{s}\right)}^{T})\cdot g_{d}\\ & \overset{\left(13\right)}{=}(g_{s}^{T}\cdot D_{j}+r\cdot {\left(a_{s}\cdot w\right)}^{T})\cdot g_{d}\\ & =(g_{s}^{T}\cdot D_{j}+r\cdot (a_{s}\cdot w^{T}))\cdot g_{d}\\ & =(g_{s}^{T}\cdot D_{j})\cdot g_{d}+r\cdot a_{s}\cdot (w^{T}\cdot g_{d})\\ & \overset{\left(14\right)}{=}(g_{s}^{T}\cdot D_{j})\cdot g_{d}+\underset{R}{\underset{︸}{r\cdot a_{s}\cdot a_{d}}} \end{array}$$

Recall that *a*_*s*_ and *a*_*d*_ are non-zero elements, and *r* is distributed uniformly over *F*_*q*_. Therefore, *R* = *r* ⋅ *a*_*s*_ ⋅ *a*_*d*_, and consequently *K*_*s*,*d*_ is distributed uniformly over *F*_*q*_. Therefore, by Definition 1, the adversary gains no information about *K*_*s*,*d*_. This concludes the proof. Using Theorem 2, we next evaluate the resilience of TurboBlom. We start with defining fail(*l*), a common measure of resilience against node capture.

**Definition 2** (Resilience: fail(*l*)). *We measure resilience of a scheme by the extent to which the scheme can withstand an adversary that captures a number of nodes. In line with previous works (e.g., [23]), we quantify this measure by* fail(*l*), *which is defined as the probability that the adversary gains information (as per Definition* 1) *about the pairwise key between two random nodes* *u*_*i*_ *and* *u*_*j*_ *by capturing* *l* *randomly chosen nodes not involving* *u*_*i*_ *and* *u*_*j*_. *The probability space is determined by the above random choices, as well as the random choices made in constructing the secret matrix* *D*, *and the generator matrix* *G*.

In the remaining of this section, we analyze fail(*l*) for TurboBlom. In Theorems 3, 4, and 5, we prove that fail(*l*) can be made exponentially small in TurboBlom when the generator matrix *G* is a random matrix.

**Theorem 3**. *Let* λ *and* *n* *be positive integers*, S *be a non-empty subset of* *F*_*q*_, G *be the set of all* (λ + 1) × *n* *matrices over* S, *and* D *be the set of all* (λ + 1) × (λ + 1) *symmetric matrices over* *F*_*q*_. *Suppose TurboBlom uses random matrices* *G and D*, *uniformly distributed over* G *and* D, *respectively*.

*Then, for every integer* 1 ≤ *l* ≤ λ, *we have*

$$\text{fail}\left(l\right)\leq 1-{\left(1-\frac{1}{{\left|S\right|}^{\lambda +1-l}}\right)}^{2},$$

*where* |S| *denotes the cardinality of the set* S.

*Proof*. Suppose that the adversary has captured *l* random nodes and extracted their secret keys. Since *G* is uniformly distributed over G, we can w.l.g. assume that the nodes captured are *u*_1_, *u*_2_, …, *u*_*l*_. Let *u*_*s*_ and *u*_*d*_, *s*, *d* > *l*, be two random uncaptured nodes. Let ***g***_*i*_ be the *i*th column vector of *G*, which corresponds to node *u*_*i*_. By Theorem 2, the adversary gains no information about the pairwise key between *u*_*s*_ and *u*_*d*_ if and only if neither ***g***_*s*_ nor ***g***_*d*_ is a linear combination of ***g***_1_, ***g***_2_, …, ***g***_*l*_.

Let *l*^′^, *l*^′^ ≤ *l*, denote the maximum number of independent vectors among ***g***_1_, ***g***_2_, …, ***g***_*l*_. We can w.l.g. assume that vectors ***g***_1_, ***g***_2_, …, ***g***_*l*^′^_ are linearly independent. By performing elementary column operations, we can convert the (λ + 1)×*l*^′^ matrix [***g***_1_, ***g***_2_, …, ***g***_*l*^′^_] into a (λ + 1) × *l*^′^ matrix that contains the identity matrix *I*_*l*^′^_, that is *I*_*l*^′^_ is a submatrix of *H*. Note that ***g***_*s*_ is a linear combination of ***g***_1_, ***g***_2_, …, ***g***_*l*_ if and only if ***g***_*s*_ is in the column vector space of matrix *H*, i.e.

$$\begin{array}{c} g_{s}=\sum_{i=1}^{l^{\prime }}a_{i}\cdot h_{i}, \end{array}$$ (15)

where ***h***_*i*_ denotes the *i*th column vector of *H*. We have $a_{i}\in S$ in (15) because i) the elements of ***g***_*s*_ are in S, and ii) the identity matrix *I*_*l*^′^_ is a submatrix of *H*.

Let

$$A=\{g|g=\sum_{i=1}^{l^{\prime }}a_{i}\cdot h_{i},\;\text{for}\;\text{some}\;a_{i}\in S\},$$

and

$$B=\{g|g={\left[g_{1},g_{2},\ldots ,g_{\lambda +1}\right]}^{T},\;g_{i}\in S\}.$$

The column vector ***g***_*s*_ is uniformly distributed over B. Moreover, Eq (15) holds if and only if $g_{s}\in A$. Therefore, the probability that (15) holds is equal to

$$\begin{array}{cc} \frac{\left|A\cap B\right|}{\left|B\right|} & \leq \frac{\left|A\right|}{\left|B\right|}\\ & =\frac{{\left|S\right|}^{l^{\prime }}}{{\left|S\right|}^{\lambda +1}}\\ & =\frac{1}{{\left|S\right|}^{\lambda +1-l^{\prime }}}\\ & \leq \frac{1}{{\left|S\right|}^{\lambda +1-l}} \end{array}$$

Thus, the probability that ***g***_*s*_ is a linear combination of ***g***_1_, ***g***_2_, …, ***g***_*l*_ is at most $\frac{1}{{\left|S\right|}^{\lambda +1-l}}.$

Similarly, we can show that probability that ***g***_*d*_ is a linear combination of ***g***_1_, ***g***_2_, …, ***g***_*l*_ is at most $\frac{1}{{\left|S\right|}^{\lambda +1-l}}$. Therefore, the probability that either ***g***_*s*_ or ***g***_*d*_ is a linear combination of ***g***_1_, ***g***_2_, …, ***g***_*l*_ is at most

$$1-{\left(1-\frac{1}{{\left|S\right|}^{\lambda +1-l}}\right)}^{2}.$$

**Example 4**. *Suppose TurboBlom uses a random generator matrix with elements from the set* S *defined in* (5), *over* *F*_*p*_, *where* *p* *is the Mersenne prime* 2^127^ − 1. *By Theorem* 3, *we have*

$$\text{fail}\left(l\right)\leq 1-{\left(1-\frac{1}{{\left|S\right|}^{\lambda +1-l}}\right)}^{2}.$$

*By Remark* (2), *the set* S *has* 2*n* + 1 *distinct elements, where* *n* = 127. *Therefore*, |S|=2n+1=2·127+1=255. *Thus, by Theorem* 3, *we get*

$$\begin{array}{cc} \text{fail}(\lambda -2) & \leq 1-{\left(1-\frac{1}{{\left|S\right|}^{\lambda +1-(\lambda -2)}}\right)}^{2}\\ & =1-{\left(1-\frac{1}{255^{3}}\right)}^{2}\\ & \approx 1.21\cdot 10^{-7}. \end{array}$$

*This shows that TurboBlom provides a high level of resilience even when* λ − 2 *nodes are captured. Recall that the Blom scheme is fully resilient up to* λ *node captures*.

#### The case of zero-one matrices

By Theorem 3, TurboBlom can achieve a high resilient against node capture if |S| is large enough (e.g., |S|=255 as in Example 4). An interesting question is whether TurboBlom can still achieve a high level of resilience if it uses a random zero-one generator matrix (for which |S|=2). A positive answer means that we can further speed up TurboBlom. This is for two reasons. First, each zero in the generator matrix—which occurs with probability 50% in a random zero-one matrix with uniform distribution—eliminates not only one field multiplication but also one field addition (because adding a number to zero is trivial). Second, if we use a zero-one generator matrix, we do not even need shift operations as multiplication by 1 is trivial.

In the following, we show the positive news that zero-one matrices can result in a fail(*l*) that is exponentially small in λ. Informally, TurboBlom equipped with a random zero-one generator matrix achieves a high resilience against node capture if λ is large enough (e.g., λ ≥ 30).

**Theorem 4**. *Let* λ *and* *n* *be positive integers*, G *be the set of all* (λ + 1) × *n* *zero-one matrices, and* D *be the set of all* (λ + 1) × (λ + 1) *symmetric matrices over* *F*_*p*_, *where* *p* > (λ + 1)! *is a prime number. Suppose TurboBlom uses random matrices* *G and D*, *uniformly distributed over* G *and* D, *respectively*.

*Then, for every integer* *l*, 1 ≤ *l* ≤ min{λ, *n* − 2}, *we have*

$$\begin{array}{c} \begin{array}{c} \text{fail}(l)\leq 2\cdot {\left(\frac{1}{2}+o\left(1\right)\right)}^{\lambda +1} \end{array} \end{array}$$ (16)

*Proof*. Suppose the adversary has captured *l*, *l* ≤ λ, random nodes and extracted their secret keys. Since *G* is uniformly distributed over G, we can w.l.g. assume that the nodes captured are *u*_1_, *u*_2_, …, *u*_*l*_. Let *u*_*s*_ and *u*_*d*_ be two random uncaptured nodes (i.e., *l* < *s*, *d* ≤ *n*). Let ***g***_*i*_ be the *i*th column vector of *G* (i.e., the column of *G* that corresponds to node *u*_*i*_). By Theorem 2, the adversary gains no information about the pairwise key between *u*_*s*_ and *u*_*d*_ if and only if neither ***g***_*s*_ nor ***g***_*d*_ is a linear combination of ***g***_1_, ***g***_2_, …, ***g***_*l*_.

Let $H_{c}$, 1 ≤ *c* ≤ λ+ 1, denote the set of all (λ + 1) × *c* zero-one matrices. Let *H* be a random matrix uniformly distributed over *H*_λ+1_. Note that the matrix *G*_*s*_ = [***g***_*s*_, ***g***_1_, ***g***_2_, …, ***g***_*l*_] is a random matrix uniformly distributed over *H*_*l*+1_. We have

$$\begin{array}{cc} & P(g_{s}\text{is}\;a\;\text{linear}\;\text{combination}\;g_{1},g_{2},\ldots ,g_{l})\\ & \leq P(\text{Rank}\left(G_{s}\right)<l+1)\\ & \leq P(\text{Rank}(H)<\lambda +1)\\ & =P((\text{det}H\;\text{mod}\;p)=0) \end{array}$$

We have

detH≤(λ+1)!

because *H* is a (λ + 1) × (λ + 1) zero-one matrix. Furthermore, by the theorem statement, we have *p* > (λ + 1)!. Therefore, we get

detH<p,

thus

(detHmodp)=0⇔detH=0.

Consequently

$$\begin{array}{cc} P((\text{det}\;H\;\text{mod}\;p)=0) & =P(\text{det}\;H=0)\\ & ={\left(\frac{1}{2}+o\left(1\right)\right)}^{\lambda +1}, \end{array}$$

where the second equation is by Theorem A of [32]. Therefore, the probability that ***g***_*s*_ (similarly ***g***_*d*_) is a linear combination of vectors ***g***_1_, ***g***_2_, …, ***g***_*l*_ is at most ${\left(\frac{1}{2}+o\left(1\right)\right)}^{\lambda +1}$. Thus, by a union bound, the probability that either ***g***_*s*_ or ***g***_*d*_ is a linear combination of vectors ***g***_1_, ***g***_2_, …, ***g***_*l*_ is at most $2\cdot {\left(\frac{1}{2}+o\left(1\right)\right)}^{\lambda +1}$, hence

$$\text{fail}\left(l\right)\leq 2\cdot {\left(\frac{1}{2}+o\left(1\right)\right)}^{\lambda +1}$$

**Example 5**. *Let* λ + 1 = 30, *that is each node is preloaded with 30 secret keys. Let* *n* *be a positive integer, and* G *be the set of all* (λ + 1) × *n* *zero-one matrices over* *F*_*p*_, *where* *p* *is a prime number. Let us set the security level to 160 bits (i.e., set the size of pairwise keys to 160 bits) by choosing* *p* *to a prime number of 160 bits, i.e.* 2^159^ < *p* < 2^160^. *Suppose TurboBlom uses a random zero-one generator matrix uniformly distributed over* G.

*We have* *p* > (λ + 1)! *in this example. Thus, by Theorem* 4, *we get that* fail(λ) *is of order* $\frac{1}{2^{30}}$. *This implies that there is only a small chance for the adversary to compromise the communication between two nodes* *u*_*s*_ *and* *u*_*d*_ *by capturing λ other nodes in the network. Note that fail(λ) is remarkably small in TurboBlom despite the use of a random zero-one matrix as the generator matrix*.

In Theorem 4, we assumed that *p* > (λ + 1)!. This does not hold in all settings. For instance, as in Example 5, we may set the security level to 160 bits (i.e. use a prime *p* of size 160 bits), but preload nodes with 50 secret keys (instead of 30 as in Example 5). In this case we have *p* < (λ + 1)! because *p* < 2^160^ < 50!. The next theorem addresses this issue by relaxing the assumption *p* > (λ + 1)!.

**Theorem 5**. *Let* λ *and* *n* *be positive integers*, G *be the set of all* (λ + 1) × *n* *zero-one matrices, and* D *be the set of all* (λ + 1) × (λ + 1) *over* *F*_*p*_, *where* *p* *is a random prime number of size* *t* *bits, that is* 2^*t*−1^ < *p* < 2^*t*^ *for an integer* *t*. *Suppose* *t* ≥ 16 (*in practice, the value of* *t* *is set much larger than 16*). *Suppose TurboBlom uses random matrices* *G and D*, *uniformly distributed over* G *and* D, *respectively*.

*Then, for every integer* *l*, 1 ≤ *l* ≤ min{λ, *n* − 2}, *we have*

$$\text{fail}\left(l\right)\leq 2\cdot \{{\left(\frac{1}{2}+o\left(1\right)\right)}^{\lambda +1}+\frac{\lambda \cdot {\text{log}}_{2}\left(\lambda +1\right)}{2^{t-1}}\}$$

*Proof*. Suppose the adversary has captured *l* random nodes and extracted their secret keys. Since *G* is uniformly distributed over G, we can w.l.g. assume that the nodes captured are *u*_1_, *u*_2_, …, *u*_*l*_. Let *u*_*s*_ and *u*_*d*_ be two random uncaptured nodes (i.e., 1 < *s*, *d* ≤ *n*). Let ***g***_*i*_ be the *i*th column vector of *G* (i.e., the column of *G* that corresponds to node *u*_*i*_). By Theorem 2, the adversary gains no information about the pairwise key between *u*_*s*_ and *u*_*d*_ if neither ***g***_*s*_ nor ***g***_*d*_ is a linear combination of ***g***_1_, ***g***_2_, …, ***g***_*l*_, that is both ***g***_*s*_ and ***g***_*d*_ are linearly independent of ***g***_1_, ***g***_2_, …, ***g***_*l*_.

Let $H_{c}$, 1 ≤ *c* ≤ λ+ 1, denote the set of all (λ+ 1) × *c* zero-one matrices. Let *H* be a random matrix uniformly distributed over *H*_λ+1_. Note that the matrix *G*_*s*_ = [***g***_*s*_, ***g***_1_, ***g***_2_, …, ***g***_*l*_] is a random matrix uniformly distributed over *H*_*l*+1_. We have

$$\begin{array}{cc} & P(g_{s}\;\text{is}\;a\;\text{linear}\;\text{combination}\;\text{of}\;g_{1},g_{2},\ldots ,g_{l})\\ & \leq P(\text{Rank}\left(G_{s}\right)<l+1)\\ & \leq P(\text{Rank}(H)<\lambda +1)\\ & =P((\text{det}\;H\;\text{mod}\;p)=0) \end{array}$$

Thus, by a union bound, we get

$$\begin{array}{c} \begin{array}{cc} & \text{fail}(l)\\ & =P(g_{s}\;\text{or}\;g_{d}\;\text{is}\;a\;\text{linear}\;\text{combination}\;\text{of}\;g_{1},g_{2},\ldots ,g_{l})\\ & \leq 2\cdot P((\text{det}\;H\;\text{mod}\;p)=0). \end{array} \end{array}$$ (17)

Let $E_{1}$, $E_{2}$ and $E_{3}$ be the following events:

$$\begin{array}{cc} E_{1}: & \text{det}\;H\;\text{mod}\;p=0\\ E_{2}: & \text{det}\;H=0\\ E_{3}: & (\text{det}\;H\neq 0)\;\wedge \;\left(p|\text{det}\;H\right). \end{array}$$

We have

$$E_{1}=E_{2}\vee E_{3},$$

Therefore, by (17), we get

$$\begin{array}{cc} \text{fail}(l) & \leq 2\cdot P(E_{1})\\ & =2\cdot P(E_{2}\vee E_{3})\\ & \leq 2\cdot P(E_{2})+2\cdot P(E_{3}), \end{array}$$

where the last inequality is by the union bound. By Theorem A of [32], we have

$$P(E_{2})={\left(\frac{1}{2}+o\left(1\right)\right)}^{\lambda +1}$$

Next, to conclude the proof, we show that

$$P(E_{3})\leq \frac{\lambda \cdot {\text{log}}_{2}\left(\lambda +1\right)}{2^{t-1}}.$$

Suppose that det *H* is non-zero and has *k* ≥ 0 different prime factors of size *t* bits. The product of *k* numbers each of size *t* bits is at least (2^*t*−1^)^*k*^ = 2^(*t*−1)*k*^. Therefore, we must have

$$\text{det}\;H\geq 2^{(t-1)k},$$

thus, by applying the log function on both sides of the above inequality, we get

$$k\leq \frac{{\text{log}}_{2}\left(\text{det}\;H\right)}{t-1}$$

Let *ζ* denote the number of prime numbers of size *t* bits. Since *p* is a random prime of size *t* bits, and det *H* has *k* different prime factors of size *t* bits, the probability that *p* is a factor of det *H* is

$$\begin{array}{c} \begin{array}{cc} P(p|\text{det}\;H) & =\frac{k}{\zeta }\\ & \leq \frac{{\text{log}}_{2}\left(\text{det}\;H\right)}{\zeta (t-1)}\\ & \leq \frac{{\text{log}}_{2}\left(\left(\lambda +1\right)!\right)}{\zeta (t-1)}\\ & \leq \frac{{\text{log}}_{2}\left({\left(\lambda +1\right)}^{\lambda }\right)}{\zeta (t-1)}\\ & \leq \frac{\lambda {\text{log}}_{2}\left(\lambda +1\right)}{\zeta (t-1)} \end{array} \end{array}$$ (18)

Due to Dusart [33], for any integer *x* ≥ 60184, we have

$$\frac{x}{\text{ln}\;x-1}\leq \pi \left(x\right)\leq \frac{x}{\text{ln}\;x-1.1},$$

where *π*(*x*) denotes the number of primes not greater than *x*. We have 2^16^ > 60184. Therefore, the number of primes of size *t* bits, *t* ≥ 16, is

$$\begin{array}{cc} \zeta & =\pi (2^{t})-\pi (2^{t-1})\\ & \geq \frac{2^{t}}{\text{ln}\;(2^{t})-1}-\frac{2^{t-1}}{\text{ln}\;(2^{t-1})-1.1}\\ & =\frac{2^{t}}{t\cdot \text{ln}\;2-1}-\frac{2^{t-1}}{(t-1)\cdot \text{ln}\;2-1.1}\\ & \geq \frac{2^{t-1}}{t-1}. \end{array}$$

Therefore, by (18) we get

$$\frac{k}{\zeta }\leq \frac{\lambda \;{\text{log}}_{2}\left(\lambda +1\right)}{2^{t-1}},$$

hence

$$\begin{array}{cc} P(E_{3}) & =\frac{k}{\zeta }\leq \frac{\lambda \cdot {\text{log}}_{2}\left(\lambda +1\right)}{2^{t-1}}, \end{array}$$

as needed.

## 5 A sample application of TurboBlom

To showcase an application of TurboBlom, we use the scheme in this section to present an efficient way for end-to-end sender authentication in RPL. We start with covering the basics of the RPL standard, and explaining our system model.

### Basics of RPL

**Fig 6:**
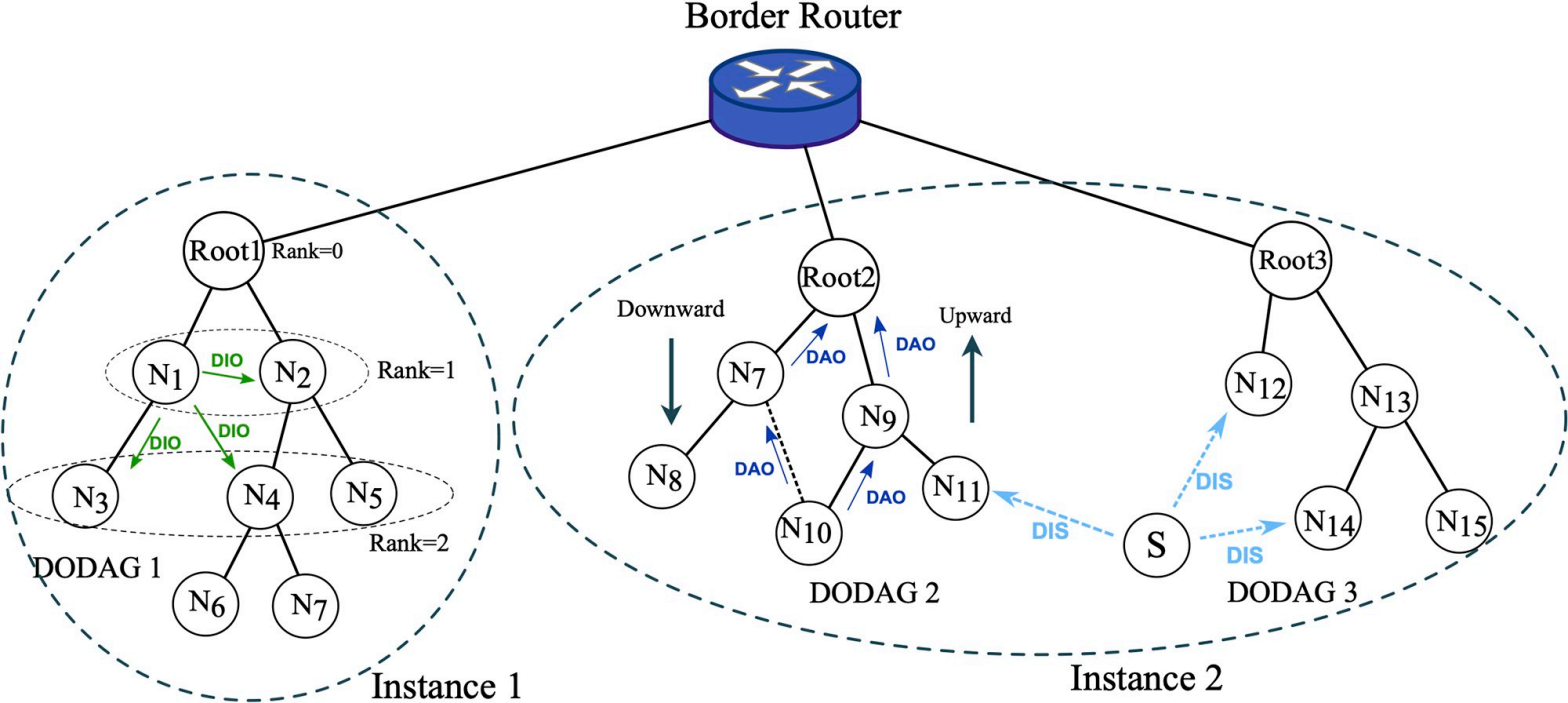
An example of RPL network with two instances and three DODAGs.

RPL is a standard routing protocol for LLNs. RPL supports three types of traffic patterns; Point-to-Multipoint (P2MP), Multipoint-to-point (MP2P), and Point-to-Point (P2P). To support these traffic patterns, RPL builds one or more Destination Oriented Directed Acyclic Graph (DODAG) each of which is uniquely identified by a set of identifier consisting DODAG ID, instance ID, and version number (Fig 6). Each DODAG has a specific node, called root, that acts as a sink for other nodes in DODAG and connect the DODAG to other network. To create and maintain the DODAG RPL uses three different control messages; DODAG Information Object (DIO), DODAG Information Solicitation (DIS), and Destination Advertisement Object (DAO). RPL control messages are ICMPv6 message with type equal to 155. The root initiates and manages the DODAG by broadcasting DIO messages over the network.

Prior to joining the network, each node listens for DIO messages. When a node receives a DIO, it can join the network. After joining the network, a node continuously advertises DIO to indicate its presence. A node can send DIS to accelerate joining the network or to recover from topology errors. RPL uses DAO to propagate nodes’ information toward the root and fill nodes’ routing tables in support of P2MP and P2P traffic.

RPL may operate in one of the following security modes:

- *unsecured mode:* In this mode, RPL does not provide a security measure. It may, however, utilize the link-layer security to protect its messages.
- *pre-installed mode:* In this mode, RPL messages are protected using pre-defined security mechanisms. secret keys are installed on nodes before they are deployed in the network. Nodes will use these preloaded keys to secure RPL messages.
- *authenticated mode:* similar to the previous mode, nodes have pre-installed keys, but these keys are used only for authenticating the nodes who want to join the network as a leaf. All nodes except the leaf nodes must obtain a second key from an authentication authority after joining the network.

**Fig 7:**
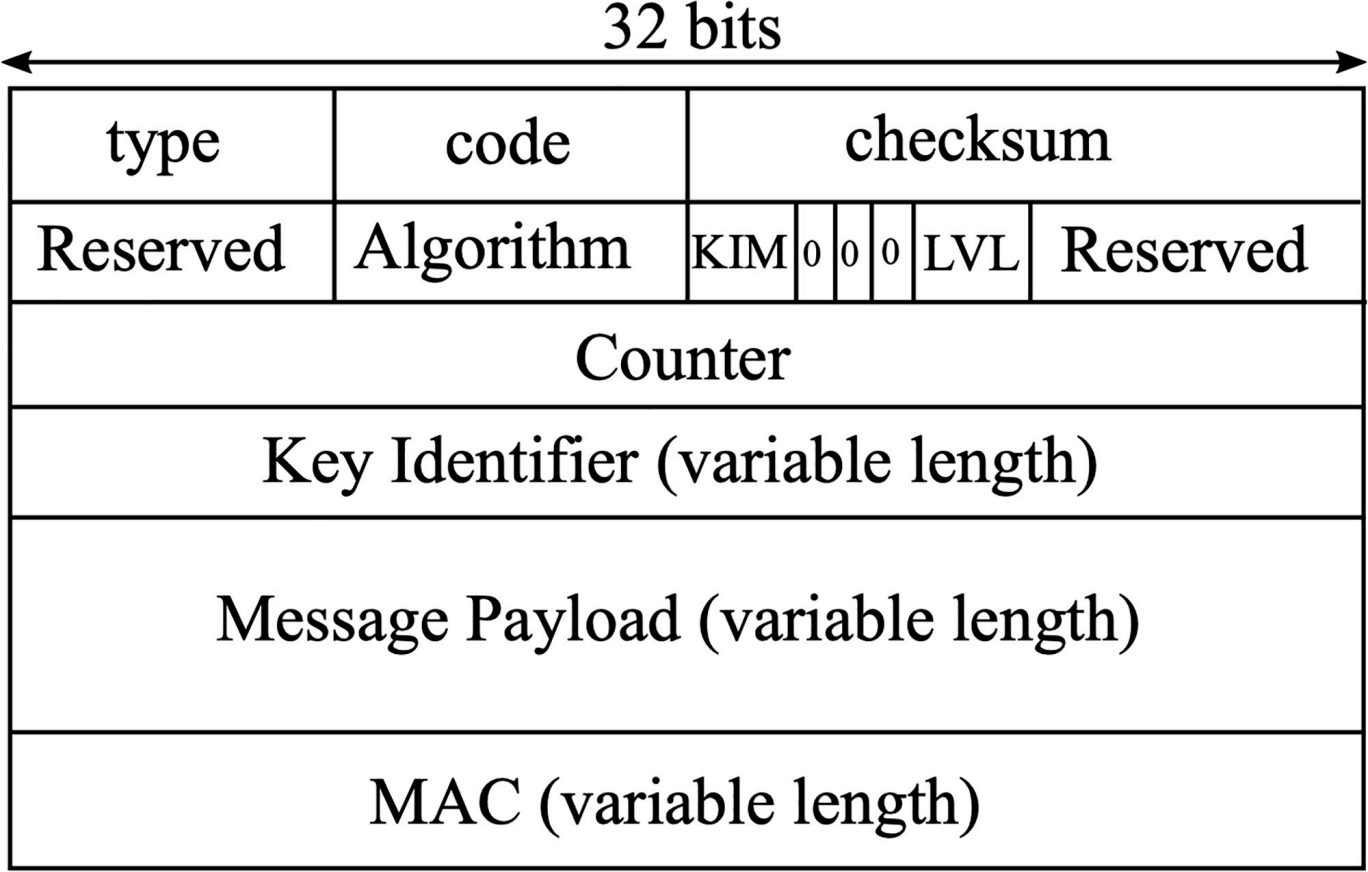
Secured ICMPv6 message format.

The general format of an RPL control message is shown in Fig 7. The ICMPv6 code field dictates the type of RPL control message: DIO, DIS, or DAO. The Algorithm field determines the algorithm used for authentication/encryption, and the LVL bits specify whether the message is only authenticated, encrypted, or both.

### Sender authentication problem in RPL

Sender authentication may be provided using symmetric-key or public-key cryptography. With public-key cryptography (via signatures), a sender can be uniquely identified, whereas with symmetric-key-based techniques, sender authenticity is only provided relative to devices in a key-sharing group. Therefore, in the absence of computationally-heavy public-key-based techniques, RPL is unable to provide fine-grained authentication, which is needed in scenarios that require group communications or in scenarios that require non-repudiation.

Many recent works have attempted to mitigate the sender’s authentication problem (or closely related problems such as the Sybil attack) in RPL networks. In a recent work, for instance, Raoof *et al.* [24] proposed a new secure mode, Chained Secure Mode (CSM), for the RPL protocol. In CSM, each node encodes its outgoing messages using a randomly generated code, and updates its neighbors with the next code. CSM only provides sender authentication between neighbors, requires a recovery mechanism, assumes that every first message truly comes from the original sender, and uses a default value of zero as the secret code for the first message from any sender. With regards to Sybil attack, current solutions (e.g., [25–29]) offer limited mitigation, can only detect a special kind of the attack, or solely detect the existence of Sybil attack in the network.

To provide sender authentication in RPL, and prevent Sybil attacks (even in the presence of small-scale capture attacks), we propose a new lightweight TurboBlom-based solution in this section. A core feature of our solution is that it prevents an adversary, with high probability, to claim the identity of an uncaptured node even when the adversary has captured and extracted the secret keys of up to λ nodes, where λ is a security parameter. We formally state this feature in Proposition 6.

In contrast to the secure CSM mode [24], our solution

- provides end-to-end sender authentication (i.e., communicating nodes do not have to be neighbors);
- does not assume that the first message is truly from the original sender;
- does not require a recovery mechanism when a message is lost (message loss can happen frequently in lossy networks such as LLNs);
- is non-interactive: a sender does not require prior interactions with a receiver in order to send an authenticated message to the receiver.

### System model

We consider an RPL network, which can support up to *n* nodes. We assume that there is a trusted entity that, prior to deployment, preloads each node with a set of λ + 1 secret keys. The trusted entity also preloads every node with public information including an IP address from a block of IP addresses [*IP*_*min*_, *IP*_*max*_] with *n* IP addresses (i.e. *IP*_*max*_ − *IP*_*min*_ + 1 = *n*), as well as the range of the block (i.e., *IP*_*min*_ and *IP*_*max*_).

We consider an adversary who has captured and extracted the secret keys of *l*, *l* ≤ λ, random nodes. The adversary can also be viewed as a coalition of *l* random malicious nodes. Our objective is to design a sender-authentication method with high resilience to the above adversary as defined below.

**Definition 3** (A Resilient Sender-Authentication Method). *Let* 0 ≤ *l* ≤ λ *be an integer*, $L\subset [IP_{min},IP_{max}]$ *be a set of* l=|L| *IP addresses selected uniformly at random, and* *IP*_*c*_ *be an IP address selected uniformly at random from* $[IP_{min},IP_{max}]\backslash L$. *Consider an adversary who has captured and extracted the secret keys of all the nodes with IP addresses in* L. *Given a sender-authentication method, let* masq(*l*) *denote the probability that the adversary is able to masquerade as* *IP*_*c*_. *Then, we call the method a resilient sender-authentication method if* masq(*l*) *is negligible, more specifically if* masq(*l*) ≤ *c*^λ^, *for a fixed constant* 0 < *c* < 1.

### TAM: A TurboBlom-based authentication mode

In this section, we introduce TAM, a lightweight TurboBlom-based pre-installed mode that provides sender authentication in RPL and is resilient to capture attacks. First, we explain how it works before nodes are deployed in the network.

#### Prior to deployment

Before nodes are deployed in the network, the trusted entity generates a random public generator matrix *G* and a random symmetric private matrix *D* over a finite field *F*_*q*_ of large order. To generate the random matrix *G*, the trusted entity uses a publicly-known random number generator (e.g., a cryptographic hash function). Since the used random number generator is publicly known, anyone can derive the column of *G* assigned to an IP address by applying the random number generator function to the IP address. This brings two advantages. First, it binds a column of *G* to an IP address. Second, nodes do not require to store any column of *G* (hence can save storage) as each column of *G* can be simply derived using the random number generator.

#### After deployment

Suppose that node *u*_*i*_ with IP address *IP*_*i*_ wants to send an authenticated message to another node *u*_*j*_, with IP address *IP*_*j*_. At the high level, *u*_*i*_ first computes *G*_:,*j*_ (the public information of *u*_*j*_) from *IP*_*j*_ by applying the random number generator to *IP*_*j*_, that is

$$\begin{array}{c} G_{:,j}=H\left(IP_{j}\right), \end{array}$$ (19)

where the function *H*(.) represents the random number generator. It then uses the inner product (3) to compute the pairwise key *K*_*i*,*j*_. After computing *K*_*i*,*j*_, *u*_*i*_ generates a HMAC of its message using the key *K*_*i*,*j*_ and concatenate the MAC to the message. Note the the sender does not require to interact with the receiver to obtain *G*_:,*j*_ because the receiver an simply calculate *G*_:,*j*_ from the IP address of the sender (i.e. from *IP*_*j*_) using (19).

**Fig 8:**
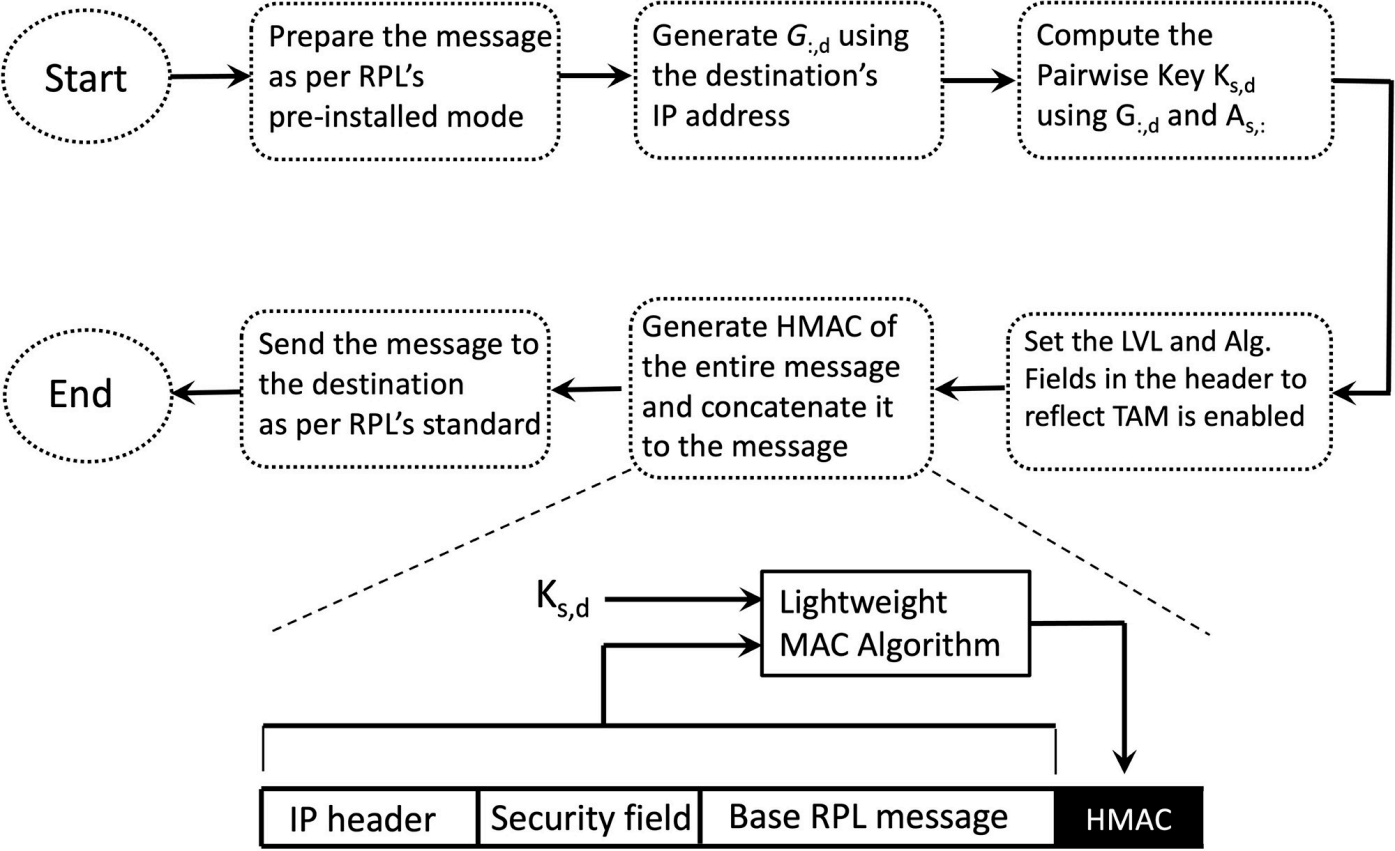
Sending a TAM authenticated packet from a source node (*s*) to a destination (*d*).

The following steps explain the above procedure in more details (see also Fig 8).

1. Prepare the message as per RPL’s pre-installed mode;
2. Generate *G*_:,*j*_ using the IP address of *u*_*j*_;
3. Compute the pairwise key *K*_*i*,*j*_ using *G*_:,*j*_ and *A*_*i*,:_, where *A*_*i*,:_ is the vector of *u*_*i*_’s secret keys. At this step, *u*_*i*_ can optionally cache *K*_*i*,*j*_ for later communications with *u*_*j*_;
4. Set the LVL and Algorithm fields in the header of ICMPv6 message (Fig 7) to reflect that TAM authentication is enabled;
5. Generate the HMAC of the entire message
  $$\text{HMAC}=\text{Hash}(K_{i,j},message),$$
  using a proper lightweight MAC algorithm such as light-MAC [34], and concatenate the MAC to the message;
6. Send the packet to the destination *u*_*j*_ as per the RPL standard.

**Fig 9:**
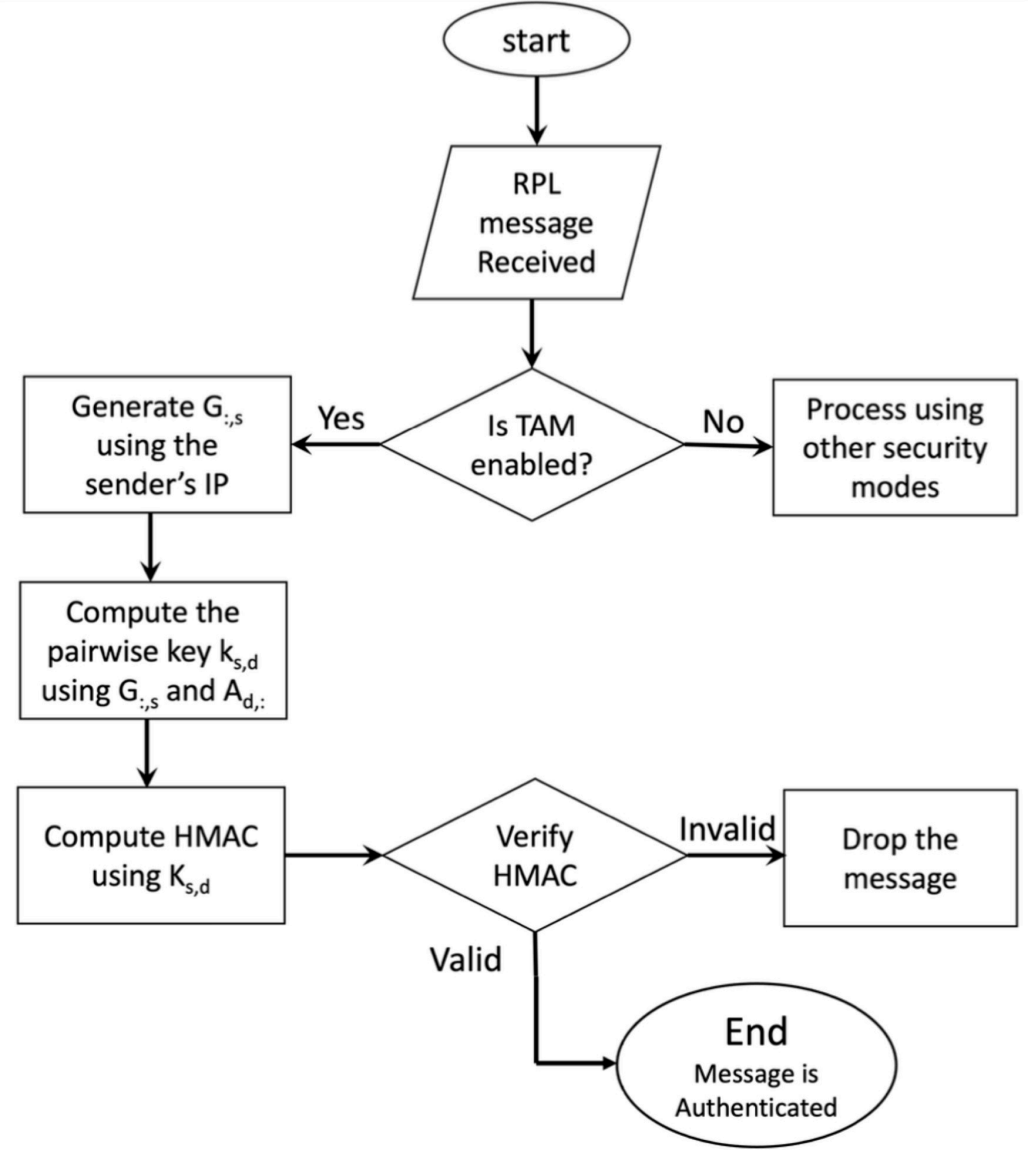
The procedure of receiving an authenticated packet in a destination node (*d*) from a source node (*s*).

At the receiver side, node *u*_*j*_ first checks the header of the received message. If the TAM mode is enabled, *u*_*j*_ checks if the sender’s IP address (which exists in the header of the received packet) is within the range [*IP*_*min*_, *IP*_*max*_]. If so, *u*_*j*_ generates *G*_:,*i*_ using the IP address of the sender. It then computes the pairwise key, *K*_*j*,*i*_ = *K*_*i*,*j*_, generates the HMAC of the received message, and compares it to the HMAC embedded in the message. If the two HMACs are equal, *u*_*j*_ accepts the message as authenticated; otherwise, *u*_*j*_ drops the message. The above procedure is illustrated in Fig 9. Note that the procedure is non-interactive, does not assume that the first message truly comes from the sender, and does not assume that the sender and receiver are neighbors.

The following proposition shows that TAM is a resilient sender-authentication method as defined in Definition 3.

**Proposition 6**. *TAM is a resilient sender-authentication method*.

*Proof*. Let *G* denote the generator matrix of the TurboBlom used in TAM. We have

$$\begin{array}{c} \begin{array}{cc} \text{fail}(l) & =1-{\left(1-\epsilon \right)}^{2}\\ & =2\epsilon -\epsilon^{2}\\ & \geq \epsilon , \end{array} \end{array}$$ (20)

where *ϵ* is the probability that the column of *G* corresponding to *IP*_*c*_ is a linear combination of the columns of *G* that correspond to the IP addresses in L (*IP*_*c*_ and L are defined in Definition 3). By definition, we have masq(*l*) = *ϵ*, thus substituting *ϵ* with masq(*l*) in (20) we get

$$\begin{array}{c} \text{masq}(l))\leq \text{fail}(l). \end{array}$$ (21)

Therefore, if we use a random zero-one matrix *G*, by Theorem 4 we get fail(*l*) ≤ *c*^λ^ for a constant 0 < *c* < 1, which by (21) implies masq(*l*) ≤ *c*^λ^ for a constant 0 < *c* < 1.

**Example 6**. *Consider a network which can support up to 1024 nodes (i.e., the network has been allocated a block with 1024 IP addresses). Suppose we use TurboBlom equipped with a random zero-one generator matrix. Let* λ + 1 = 50, *that is each node is pre-laoded with 50 secret keys. By Theorems* 4 *and* 5, $\text{fail}\left(l\right)\approx \frac{1}{2^{50}}$ *for any* *l* ≤ λ. *Therefore, by the union bound, the probability that an adversary who has captured* *l* < 50 *random nodes is able to masquerade as any valid IP in the block of 1024 IPs (other than those 50 IP addresses that belong to the captured node) is at most*

$$\begin{array}{cc} \left(1024-50)\cdot \text{masq}(l)\right) & \leq 1024-50)\cdot \text{fail}(l))\\ & \approx 10^{-13} \end{array}$$

*for any* *l* ≤ λ. *Therefore, an adversary who has captured less than 50 random nodes has an extremely low chance* (≈10^−13^) *of being able to masquerade as any valid IP address that does not belong to the captured nodes*.

## 6 A numerical case study

To confirm our analytical results, we evaluated the resilience of TurboBlom in a practical setting, where we assume a network with up to *n* = 1000 devices, each capable of storing λ + 1 = 30 secret keys. We set the order of the finite field to 160 bits, and constructed the generator matrix *G* randomly by choosing its elements from S={-1,0,1} uniformly at random. The choice of S is because it reduces all operations in the key generation into simple additions and subtractions, yet it provides higher resilience than the case S={0,1}. In simple words, the use of S={-1,0,1} in practice is more justified than the use of S={0,1}, as the former provides higher resilience at virtually the same computational cost as the latter. The simulation setting parameters are summarized in Table 2.

**Table 2:** Simulation setting parameters.

Parameter	Value	Rationale
No. of devices	*n* ≤ 1000	Covers most applications
No. of stored keys	λ + 1 = 30	A small value of λ to showcase the high resilience of TurboBlom.
Order of finite field	160 bits	To set the security bits to 160 bits
The set S	S={-1,0,1}	Provides higher resilience than S={0,1} at virtually the same computational cost

We simulated the capturing attack by selecting λ = 29 columns of *G* uniformly at random and checking if the capture of these 29 nodes (corresponding to the selected columns) would compromise the key of any of the uncaputured nodes. We repeated the above process 100, 000 times. The above numerical analysis took more than 240 hours on a Microsoft Azure sever with 64 virtual CPUs and 512 GB RAM, yet it was unable to find any set of 29 nodes whose capture results in the compromise of a pairwise key between uncaptured nodes. This is in line with our analytical results which showed that the chance of an adversary to compromise the pairwise key between two uncaptured nodes is extremely low when the adversary captures up to λ = 29 random nodes.

## 7 Conclusion and future work

In this paper, we introduced TurboBlom, a variant of the well-known Blom key predistribution scheme. TurboBlom is significantly lighter that the Blom scheme as it does not require any field multiplications. Yet, similar to the Blom scheme, TurboBlom 1) guarantees that any two nodes can generate a pairwise key, and 2) is highly resilient against node capture attack. This is notable as the Blom scheme provides the highest level of resilience against small-scale node capture attacks. The above features of TurboBlom make it suitable for computationally-constrained devices which exist in abundance in IoT, for instance as part of RPL networks. Finally, to showcase TurboBlom, we proposed TAM, a lightweight and TurboBlom-based secure mode that enables sender authentication in RPL networks. The proposed authentication method is highly resilient to node capture/collusion thanks to the proven resilience of TurboBlom.

One interesting future research direction is to improve the result of Theorem 3. Theorem 3 already establishes a lower bound on the resilience of the TurboBlom. By Theorems 4 and 5, we believe that there is substantial room for enhancing this lower bound. Another interesting direction is to analyze node capture attacks in a setting where the attacker can selectively, rather than randomly (as typically done in the literature), capture nodes.
